# Development of a PANoptosis‐Related Pathomics Prognostic Model in Ovarian Cancer: A Multi‐Omics Study

**DOI:** 10.1111/jcmm.70958

**Published:** 2025-11-24

**Authors:** Yangyang Zhang, Mengqi Fang, Xuanyu Wang, Zhiwei Ying, Shufan Jiang, Yangyuxiao Lu, Keren He, Shaocong Mo, Fangfang Tao, Ping Lü

**Affiliations:** ^1^ Shanghai Medical College Fudan University Shanghai China; ^2^ The First Affiliated Hospital of Zhejiang Chinese Medical University, Zhejiang Provincial Hospital of Chinese Medicine Zhejiang Chinese Medical University Hangzhou China; ^3^ College of Traditional Chinese Medicine Tianjin University of Traditional Chinese Medicine Tianjin China; ^4^ College of Electronic Information and Electrical Engineering Shanghai Jiao Tong University Shanghai China; ^5^ Department of Pathology, Shanghai Medical College Fudan University Shanghai China; ^6^ Department of Digestive Diseases, Huashan Hospital Fudan University Shanghai China; ^7^ Department of Immunology and Microbiology, Basic Medical College Zhejiang Chinese Medical University Hangzhou Zhejiang China; ^8^ Department of TCM Taizhou First People’s Hospital Hangzhou Zhejiang China

**Keywords:** multi‐omics analysis, ovarian cancer, PANoptosis, pathomics prognostic model, *STAT4*

## Abstract

Ovarian cancer (OC) is a high‐mortality gynaecological malignancy, and the role of PANoptosis, a comprehensive cell death mechanism, in its prognosis remains unexplored. This study aims to clarify it, potentially guiding OC diagnosis and treatment. We analysed the ovarian data from TCGA and GTEx, and the GSE184880 scRNA‐seq dataset from GEO. Spatial data and pathological images were sourced from the 10X Genomics website and GDC Portal. Features were extracted using CellProfiler and ResNet‐50, and a PANoptosis‐related pathomics prognostic model (PANPM) powered by deep learning was developed. The PANoptosis‐related hub gene *STAT4* potentially served as a protective factor for patients with OC. A better prognosis in OC was found linked to higher PANoptosis. The PANPM, manifesting distinct advantages for clinical application by accurately extracting pathological features, performed excellently in validation and the high‐risk group indicated a poor prognosis. Additionally, *STAT4* ^+^ T cells may inhibit OC, by activating the PANoptosis of epithelial cells through TNFSF12‐TNFRSF12A and TNF‐TNFRSF1A, which sheds light on potential therapeutic interventions involving *STAT4* ^+^ T cells.

## 1 Introduction

Ovarian cancer (OC) is a severe malignancy of the female reproductive system, with a 5‐year relative survival rate of less than 50% [1]. According to the National Comprehensive Cancer Network (NCCN) guidelines for OC, surgery and chemotherapy are currently the preferred treatment options [2]. Due to the indistinct early symptoms and the lack of systematic opportunistic screening methods [3], more than two‐thirds of OC patients are diagnosed at an advanced stage, further complicating treatment strategies [4]. Notably, the high recurrence rate of OC [5] and the accompanying physical and mental impact [6] have brought significant burdens to the lives of OC patients. Therefore, searching for reliable prognostic markers and utilising new intervention measures to improve prognosis will bring hope for OC patients.

One important factor in cancer development is the ability of cancer cells to evade programmed cell death (PCD). Therefore, inducing cell death is crucial to cancer therapy [7]. Recently, PCD has received widespread attention, especially the compelling bond between PCD and cancers [8]. Advancements in research have shed light on the complex interplay among multiple PCD pathways [9]. Therefore, in 2019, Malireddi et al. proposed the concept of PANoptosis, an inflammatory PCD pathway regulated by the PANoptosome complex, which is a novel death mode characterised by pyroptosis, apoptosis, and necroptosis simultaneously [10]. When one or more PCD pathways in humans are inhibited, PANoptosis can be ushered in as an alternative method to induce cell death [11]. Hence, the activation of PANoptosis serves as an effective defence mechanism, while excessive activation or suppression can trigger various diseases [12]. The implication of PANoptosis in tumours is also gradually being recognised [13]. Although its specific regulatory mechanisms in cancer are elusive, PANoptosis has promising prospects as a potential intervention target.

Prognostic models that incorporate various predictors can be used to estimate the risk of disease development and prognosis [14], which can minimise potential harm and costs based on guiding clinical practice. In the field of oncology, prognostic models have found widespread use [15]. Recently, there's been an application of models focused on PANoptosis in cancer scenarios [16], offering substantial assistance to clinicians and patients. Although there are prognostic models related to pyroptosis [17] and necroptosis [18] in OC, the role of PANoptosis in OC has not been explored in the existing findings.

**FIGURE 1:**
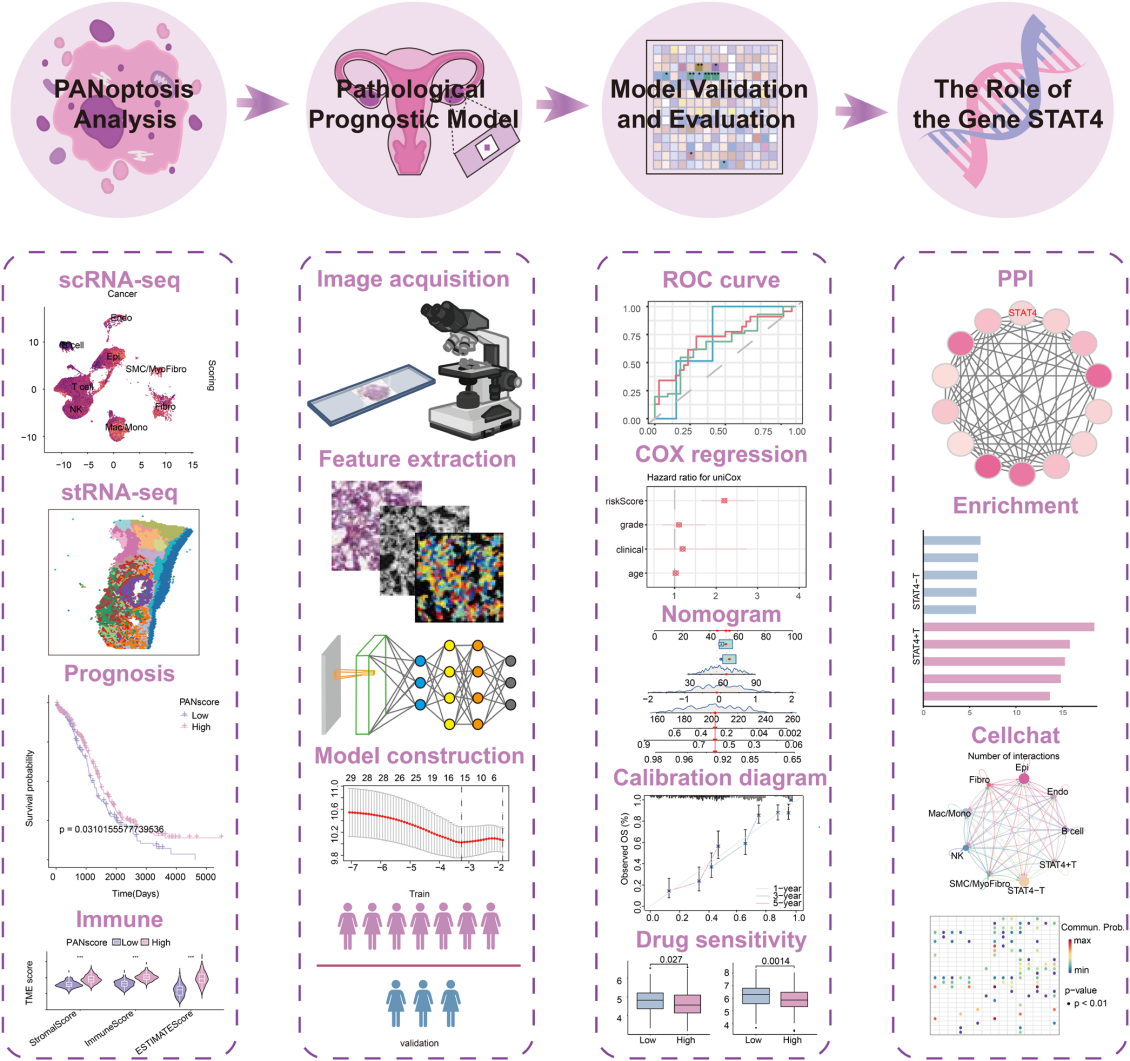
Flow diagram of study design.

Our study explored the intricate association between PANoptosis and OC utilising a multi‐omics approach and illuminated its preponderant prognostic role from a fresh perspective through pathomics insights (Figure 1). Our findings are anticipated to serve as a reliable reference for clinical prognosis in OC and pave the way for further investigation into potential immunotherapeutic targets beneficial for OC patients.

## 2 Methods

### 2.1 Data Acquisition

In this study, bulk RNA‐seq data from 421 OC patients and corresponding clinical details were sourced from The Cancer Genome Atlas (TCGA) database (https://www.cancer.gov/ccg/research/genome‐sequencing/tcga) [19]. The normal ovarian tissue data (*N* = 89) were acquired from the Genotype Tissue Expression Project (GTEx) database (https://www.gtexportal.org/home/index.html) [20]. Single‐cell RNA‐sequencing (scRNA‐seq) data (GSE184880) were accessed from the Gene Expression Omnibus (GEO, https://www.ncbi.nlm.nih.gov/geo/) [21]. We also utilised the original count matrix, histological images and spatial location data of human OC on the 10X Genomics website (https://support.10xgenomics.com/spatial‐gene‐expression/datasets). Furthermore, we downloaded and screened whole‐slide images (WSIs) of haematoxylin and eosin (H&E) stained OC patient samples (*N* = 518) from the Genomic Data Commons portal (GDC‐portal, https://portal.gdc.cancer.gov/). The H&E images and clinical information of the Prostate, Lung, Colorectal and Ovarian (PLCO) cohort and Cancer Imaging Archive (TCIA) cohort for external validation were collected from the PLCO Cancer Screening Trial and the TCIA (https://www.cancerimagingarchive.net/collection/ovarian‐bevacizumab‐response/) [22]. The criteria for inclusion and exclusion can be seen in Figure S1. Lastly, we compiled the PANoptosis‐associated gene list for further analyses by merging genes involved in pyroptosis, apoptosis, and necroptosis [23].

### 2.2 Image Segmentation and Image Feature Extraction

To facilitate feature extraction, we segmented WSIs employing the Python library ‘histolab’ [24]. The WSIs were segmented into sub‐images with 512 × 512‐pixel dimensions, extracted from images at 20× magnification. Using histolab's tile scoring framework, the top 20 most informative images were chosen. The pathologist then reviewed these images, excluding any of poor quality (contaminated, blurry, or with more than 50% white space). We meticulously selected a maximum of nine sub‐images per patient for further analysis.

We utilised the open‐source image analysis software CellProfiler (v4.2.6) [25] developed by the Broad Institute, for quantitative extraction of pathomics features from the selected pathology patches. Based on the ‘Unmix Colors’ module, we separated H&E‐stained images and converted them into haematoxylin‐stained and eosin‐stained grayscale images [26]. The H&E‐stained images were also converted to grayscale images using the ‘ColorToGray’ module [27]. Two separate measurements were conducted. The initial measurement generated 296 summary features for the three image types. The secondary measurement involved an extensive exploration of the haematoxylin images. For this, we identified primary and secondary objects, which were then measured. Their mean, median, and standard deviation values were recorded as the research characteristics. This resulted in extracting 844 pathomics features, which were then aggregated by calculating the mean values of every extracted tile in each WSI. The specifics of the feature extraction method are further detailed in Figures S2 and S3.

ResNet‐50 was employed to extract deep‐learning features of pathomics [28]. Specifically, the pre‐trained ResNet‐50 model was utilised to extract feature embeddings of image patches, achieved through spatial maximal pooling following the fourth residual block. We concatenated the derived feature vector for each patch, resulting in 2048 dimensions. Following this, we combined the 1140 features derived from CellProfiler with the 2048 features gleaned via ResNet‐50 for subsequent model construction.

### 2.3 Development of a PANoptosis‐Related Pathomics Prognostic Model (PANPM)

We employed the Spearman correlation analysis (*p* < 0.05) and an Elastic network (alpha = 0.02) to identify 317 features related to PANoptosis. To establish a consensus on PANoptosis‐related genes with high accuracy and stability, we developed a comprehensive approach by integrating nine machine‐learning algorithms and 39 algorithm combinations. These integrated algorithms included a range of techniques, such as support vector machine (SVM), least absolute shrinkage and selection operator (Lasso), gradient boosting machine (GBM), random forest (RSF), stepwise Cox, Ridge, CoxBoost, super partial correlation (SuperPC), and partial least squares with Cox regression (plsRcox). The procedure for generating the signatures involved the following steps: (a) Using univariate Cox analysis, we identified 30 PANoptosis‐related pathomics prognostic features (Table S1). (b) Subsequently, 39 algorithm combinations were applied to the prognostic PANoptosis features to fit prediction models using repeated random sub‐sampling cross‐validation (RRS‐CV) in the TCGA cohort. (c) All models were further cross‐validated using two independent datasets (PLCO and TCIA). (d) For each model, Harrell's concordance index (C‐index) was calculated across all validation datasets, and the model with the highest average C‐index was deemed optimal.

Subsequently, through Lasso analysis, we ascertained 15 pivotal prognostic features in constructing our PANPM. Leveraging the median risk score from the model, we categorised OC patients into the high‐ and low‐risk groups. A prognostic assessment was followed using the Kaplan–Meier curve. Moreover, to evaluate the predictive performance of our model, we used the receiver operating characteristic (ROC) curve along with a nomogram that incorporated factors such as age, clinical stage, pathological grade, and risk score. The calibration plot, cumulative hazard, and decision curve analysis (DCA) related to the nomogram were also generated. All analyses were conducted in R (version 4.3.1). Key packages included caret (6.0‐94), glmnet (4.1‐8), randomForest (4.7‐1.1), xgboost (1.7.5), survival (3.8‐3), and plsRcox (1.7.7).

### 2.4 scRNA‐Seq Analysis

We analysed scRNA‐seq data using the ‘Seurat’ R package (v4.3.0) [29]. Cells of inferior quality were eliminated, defined by less than 200 genes or exceeding 10,000 being expressed or having more than 50% of unique molecular identifiers (UMIs) originating from the mitochondrial genome. Using ‘SCTransform’, we normalised the data by constructing a regularised negative binomial model of gene expression and identifying characteristics with high variance [30]. Afterward, the ‘RunPCA’ function was used for principal component analysis (PCA) on the highly variable genes, choosing the top 30 principal components (PCs). The ‘harmony’ R package (v1.0) was applied to rectify technical differences among the samples [31]. Furthermore, we used the uniform manifold approximation and projection (UMAP) approach to display the annotated cells.

Five algorithms were performed to calculate PANoptosis‐related gene activity for each cell in OC, including AddModuleScore, ssGSEA, AUCell, UCell, and singscore [32]. Subsequently, we performed Gene Ontology (GO) to clarify the role of *STAT4* in OC further using the ‘clusterProfiler’ R package (v4.9.3) [33]. Gene set enrichment analysis (GSEA) was used to identify the enriched biological processes in *STAT4* ^+^ T cells compared to *STAT4* ^−^ T cells. Moreover, we utilised the ‘CellChat’ R package (v.1.6.1) to examine the communication among various cell subtypes [34]. The ‘CellChat’ package enabled us to identify interactions between ligands and receptors in different cell subsets by examining their gene expression profiles. The ‘scMetabolism’ R package (v.0.2.1) was utilised to conduct metabolic analysis on various cellular subpopulations [35]. By implementing the vision algorithm, the ‘scMetabolism’ package measures the metabolic pathway activity of every cell subpopulation in the single‐cell expression matrix.

### 2.5 Spatial Transcriptomics (ST) Data Analysis

To better understand the spatial distribution of tumour clusters in OC, we utilised ST data in our analysis. Dimensionality reduction and clustering were carried out by applying ‘RunPCA’, ‘FindNeighbors’, and ‘FindClusters’ to the top 30 principal components. Using the ‘SpatialFeaturePlot’ function in ‘Seurat’, we produced plots showing the expression of spatial features. Furthermore, we employed reverse compositional transcriptomics deconvolution (RCTD) to determine the underlying cell types in each spot [36]. Additionally, we employed the ssGSEA algorithm in ST data to identify cell types with enhanced activity in PANoptosis. We utilised scRNA‐seq cell type profiles to determine spatial interactions to perform SPOTlight deconvolution on our spatial transcriptome [37].

### 2.6 Immune Response, Drug Sensitivity, and the Tumour Immune Microenvironment Analysis

We used the TIDE web tool to predict the responsiveness of the high‐ and low‐risk groups to immunotherapy, and lower TIDE scores indicated better immunotherapy efficacy [38]. Spearman analysis was also used to evaluate the correlation between risk score and essential immune check‐related genes. In addition, we applied SubMap to calculate the expression similarity between patients in the high‐ and low‐risk groups and patients who responded and did not respond to immune checkpoints, thus inferring the efficacy of immunotherapy [39]. We used the ‘oncoPredict’ package to compare drug sensitivity between the high and low‐risk score groups [40]. Furthermore, the ‘IOBR’ R package served as a convenient platform for activating various immune infiltration methods [41]. Using this package, we deployed MCPcounter, quanTIseq, Xcell, ESTIMATE, EPIC, and CIBERSORT algorithms to compute the infiltration scores of different immune cells [42].

### 2.7 Identification of the Hub Gene *STAT4*

We conducted a Spearman correlation analysis to identify 180 PANoptosis‐related genes strongly associated with pathological features (*p* < 0.001), as detailed in Table S2. Protein–protein interactions (PPIs) contribute significantly to cell biology research and serve as an essential prerequisite for systems biology studies. We located proteins within PPI networks based on their interactions with other proteins, suggesting their potential functions. Using the STRING repository to delineate functional and physical interactions, we constructed PPI networks from genes linked to pathological features (https://string‐db.org/). Subsequently, the PPI network underwent analysis through the MCODE plugin using default parameters (degree cutoff ≥ 2, node score cutoff ≥ 2, K‐core ≥ 2, and max depth = 100). As a result, we extracted a subnetwork comprising 14 hub genes with top scores (Table S3). Further, through univariate Cox analysis, we identified the *STAT4* gene with a strong correlation to prognosis.

### 2.8 Multi‐Omics External Validation

To further substantiate the role of *STAT4* ^+^ T cells in activating PANoptosis in epithelial cells of OC patients, we integrated diverse datasets employing single‐cell, Visium, and Xenium spatial transcriptomics approaches.

### 2.9 External Single‐Cell Datasets

A total of 18 external single‐cell datasets were employed, collectively creating a comprehensive cell atlas for OC patients. To ensure data integrity, cells identified as low‐quality or germline in the original studies were excluded. Only cells meeting strict criteria (500–8000 detected genes, 1000–100,000 gene counts, and less than 20% mitochondrial gene content) were retained. Samples with fewer than 50 high‐quality cells were discarded. Following this filtration, we analysed gene count and mitochondrial content distributions, applying thresholds to filter out droplets with technical artefacts. Gene counts were normalised and log‐transformed using Scanpy's sc.pp.normalize_total and sc.pp.log1p functions. Highly variable genes (HVGs) were identified via scanpy.pp.highly_variable_genes with parameters n_top_genes = 2000, flavor = ‘cell_ranger’ and batch_key = ‘datasetID’. Following final dataset curation, principal component analysis (PCA) was performed on HVGs. Subsequently, dataset integration was achieved using Scanpy's BBKNN implementation with default parameters. Annotations and further integration were conducted using the Scanpy toolkit, adhering to default settings unless specified.

### 2.10 Statistical Analysis

Statistical analyses were executed utilising R software (v4.2.7), accessible at https://www.r‐project.org/. Survival data were subjected to Kaplan–Meier survival analysis. Prognostic factors that independently indicated risk were discerned using univariate and multivariate Cox regression analyses. Wilcoxon's test assessed the statistical significance of non‐normally distributed data, while the *t*‐test was used to evaluate statistical differences in normally distributed data. A *p*‐value of less than 0.05 was established as the statistical significance threshold.

## 3 Results

### 3.1 PANoptosis Analysis and Immune Infiltration Analysis in OC

**FIGURE 2:**
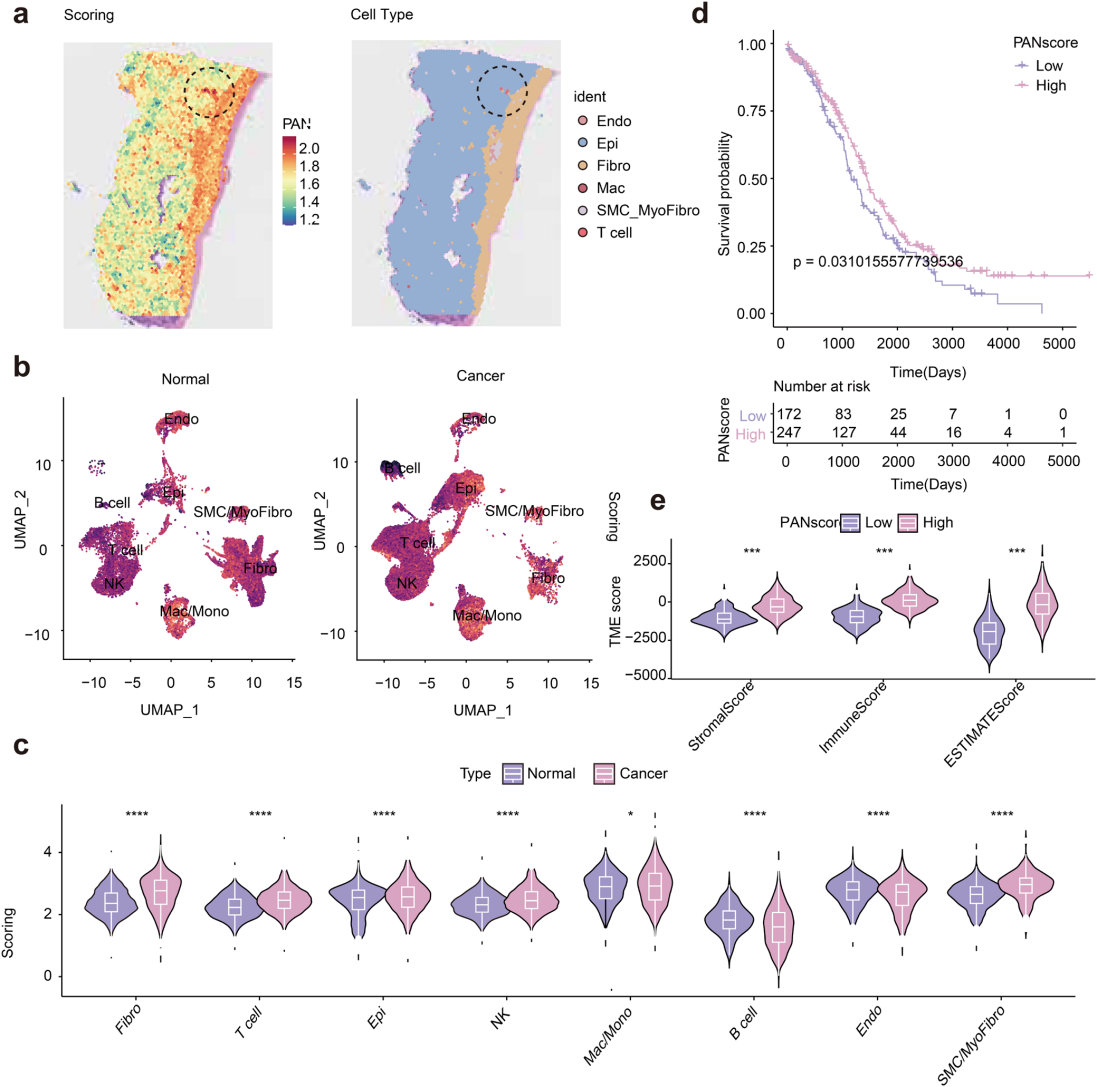
PANoptosis analysis and immune infiltration analysis in ovarian cancer (OC). (a) Spatial transcriptomic analysis of PANoptosis scores and cell distribution. (b) Enrichment scores of PANoptosis‐related genes in each cell type are shown by UMAP, with a darker purple colour having lower scores. (c) PANoptosis in different cell populations. (d) Prognosis related to PANoptosis. (e) ESTIMATE algorithm for evaluating immune infiltration levels in different PANoptosis groups. *p < 0.01; ***p < 0.001; ****p < 0.0001.

Through stRNA‐seq (Figure 2a), we revealed that the ovary of OC was mainly composed of epithelial cells and fibroblasts, with the latter demonstrating a superior level of PANoptosis. Intriguingly, elevated levels of PANoptosis were also observed in T cells. We implemented a dimensionality reduction analysis on our single‐cell data, with the resulting UMAP depicted in Figure 2b. The PANoptosis levels of various cells in normal tissues and OC tissues were also analysed (Figure 2c). It can be found that most cells have increased PANoptosis levels in OC compared to the normal, except for B cells and endothelial cells. Then, based on the different levels of PANoptosis in OC, we divided OC samples into a high PANoptosis group and a low PANoptosis group. Prognostic analysis as depicted in Figure 2d showed that the prognosis of the high PANoptosis group was significantly better than that of the low PANoptosis group (*p* < 0.05). Thus, PANoptosis was a protective factor for OC, which may be expected to improve patient prognosis in clinical practice. Moreover, there were higher Stromal Score, Immune Score, and ESTIMATE Score in the high PANoptosis group, compared with the low group (Figure 2e). More details on the role of PANoptosis in OC are presented in Figure S4.

### 3.2 Development and Validation of a PANPM

**FIGURE 3:**
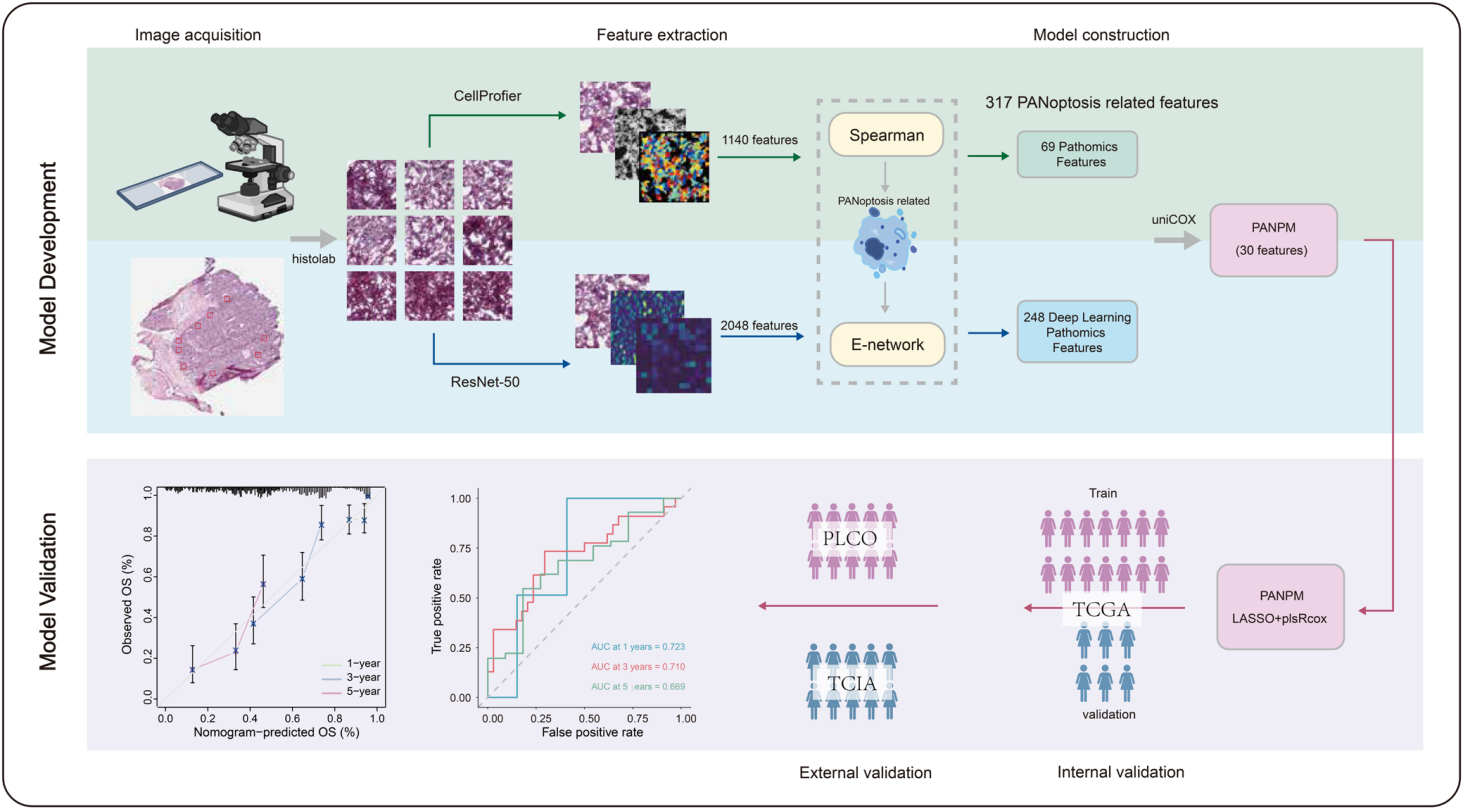
Flow diagram of development and validation of PANoptosis‐related pathomics prognostic model (PANPM).

**FIGURE 4:**
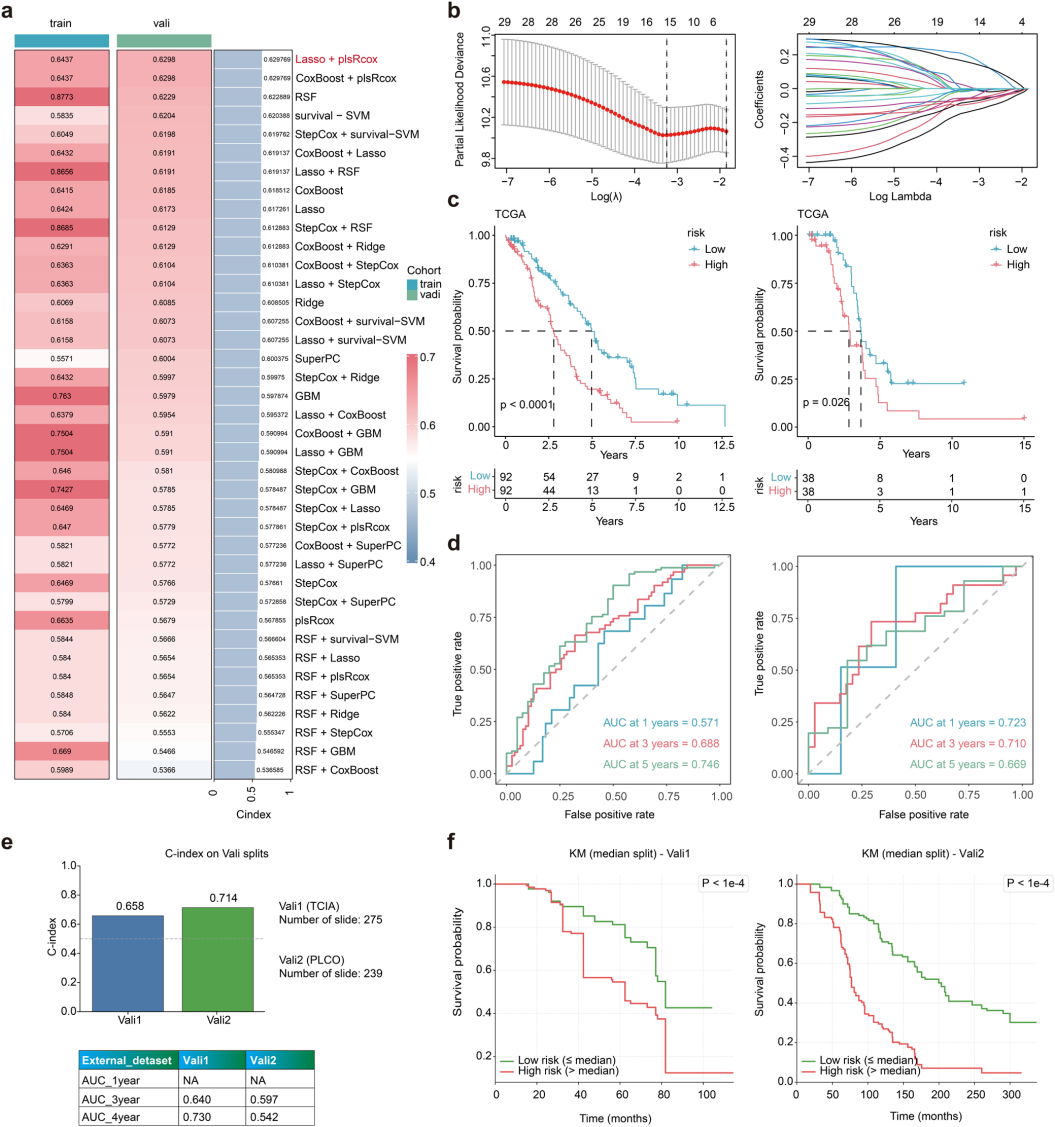
Development and validation of prognostic model. (a) A total of 39 prediction models and the Harrell consistency index (C‐index) of each model. (b) Least absolute shrinkage and selection operator (LASSO) regression analysis to screen predictive features. (c) Prognostic analysis of the train set (left) and the validation set (right). (d) Receiver operating characteristic (ROC) curves for the train set (left) and the validation set (right). (e) The C‐index of the two external validation sets (above); the AUC of two external validation sets at 1, 3, and 5 years (below). (f) Prognostic analysis of the TCIA set (left) and the PLCO set (right).

**FIGURE 5:**
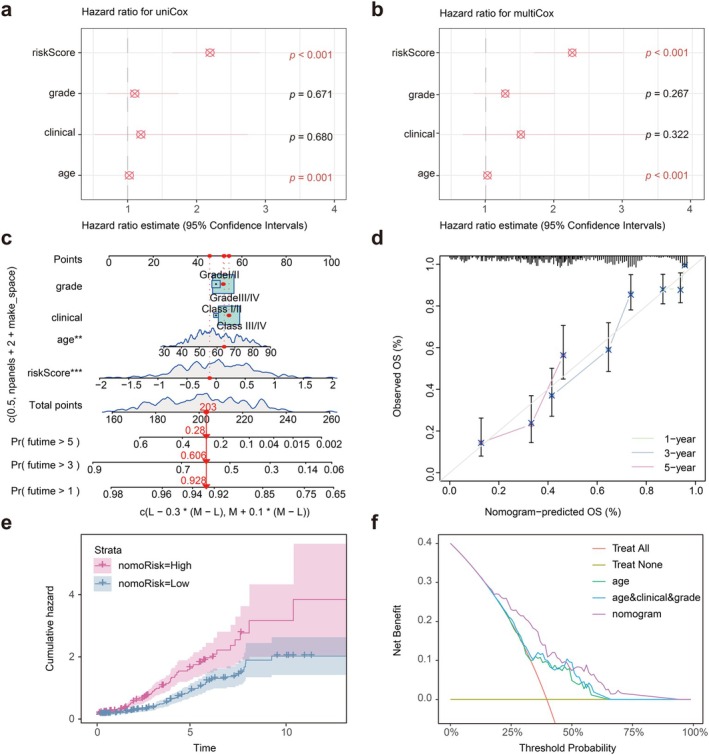
Development and validation of the nomogram. (a) Univariate Cox regression based on the risk score of the model. (b) Multivariate Cox regression based on the risk score of the model. (c) A nomogram based on risk score and clinical characteristics. (d) Calibration curves for 1‐, 3‐ and 5‐year overall survival. (e) Cumulative hazard calculated the cumulative mortality risk of OC patients in different risk groups. (f) Decision curve analysis (DCA) curve was compared over a period of 3 years for patients with OC.

Specific modalities regarding PANPM are reflected in Figure 3. A total of 39 algorithm combinations were applied to construct prognostic models. The C‐index of each model on all validation datasets was calculated, leading to the selection of Lasso and plsRcox (Figure 4a). Subsequently, we procured pathological sections from the TCGA database for screening. We employed ResNet‐50 and CellProfiler tools to extract essential features from the pathological sections for ensuing analysis. We employed ResNet‐50 and CellProfiler tools to extract essential features from the pathological sections for ensuing analysis (Figure 4b). Patients were then classified into the high‐ and low‐risk groups based on the median. As depicted in Figure 4c, the prognosis for the high‐risk group in the training set was significantly inferior to the low‐risk group (*p* < 0.0001). Comparable results were observed in the validation set, reflecting a poorer prognosis for the high‐risk group (*p* < 0.026). In addition, we used ROC curves to evaluate the model's predictive performance (Figure 4d). The results showed that the AUC of the test set at 1, 3, and 5 years was 0.571, 0.688, and 0.746. Similarly, the AUC of the validation set for 1, 3, and 5 years was 0.723, 0.710, and 0.669, respectively. In addition, we conducted external validation using the PLCO dataset and TCIA dataset, with a C‐index of 0.714 and 0.658. The AUC of the PLCO validation set at 3 and 5 years was 0.597 and 0.542. The AUC of the TCIA validation set at 3 and 5 years was 0.640 and 0.730 (Figure 4e). As depicted in Figure 4f, the prognosis of the high‐risk group was observed to be poor in both the PLCO validation set (*p* < 1e‐4) and (*p* < 1e‐4). These outcomes substantiated the excellent predictive performance of our model. Univariate and multivariate Cox analyses, recorded in Figure 5a,b, also confirmed that our risk score had a more significant negative effect than other clinical features (*p* < 0.001). We additionally analysed clinical features and risk score through a nomogram to predict 1‐, 3‐, and 5‐year survival rates in patients with OC (Figure 5c). The calibration curve confirmed that the accuracy of the nomogram was consistent with the actual situation (Figure 5d). The cumulative hazard also confirmed that the high‐risk group in the nomogram had a higher cumulative probability of death occurrence (Figure 5e). DCA indicated that the nomogram had significant superiority in clinical benefits for patients (Figure 5f).

### 3.3 Immune Infiltration Analysis and Efficacy Prediction in the High‐ and Low‐Risk Groups

We conducted an in‐depth analysis of the immune infiltration corresponding to different risk scores (Figure S6a), revealing diverse infiltration levels by different immune cells under various conditions. Nonetheless, high infiltration was predominantly observed within the high‐risk group. We further analysed the correlation between 15 pathological features and immune infiltration using CIBERSORT and Xcell (Figure S5a,b). According to Xcell, it was observed that the risk score was related to the abundance of immune cells (Figure S5c). The results also showed that the high‐risk group had higher Stromal Score, Immune Score, and ESTIMATE Score (Figure S5d).

To further explore our model's clinical value, we predicted immunotherapy efficacy according to TIDE (Figure S6b,c), consequently revealing a superior immunotherapy response within the low‐risk group. The correlation between risk score and immune check‐related genes, including *CTLA4*, *PDCD1LG2,* and *CD247*, is shown in Figure S6d. The results of SubMap indicated that the expression patterns of the low‐risk group were more similar to those of OC patients who responded to PD‐1 treatment (Figure S6e). The results of drug sensitivity analysis in Figure S6f showed that the low‐risk group had higher sensitivity to almost commonly used targeted drugs (*p* < 0.05), such as AZD8186, Selumetinib, and Dasatinib. However, the high‐risk group had a higher sensitivity to Sepantronium bromide (*p* = 0.02).

**FIGURE 6:**
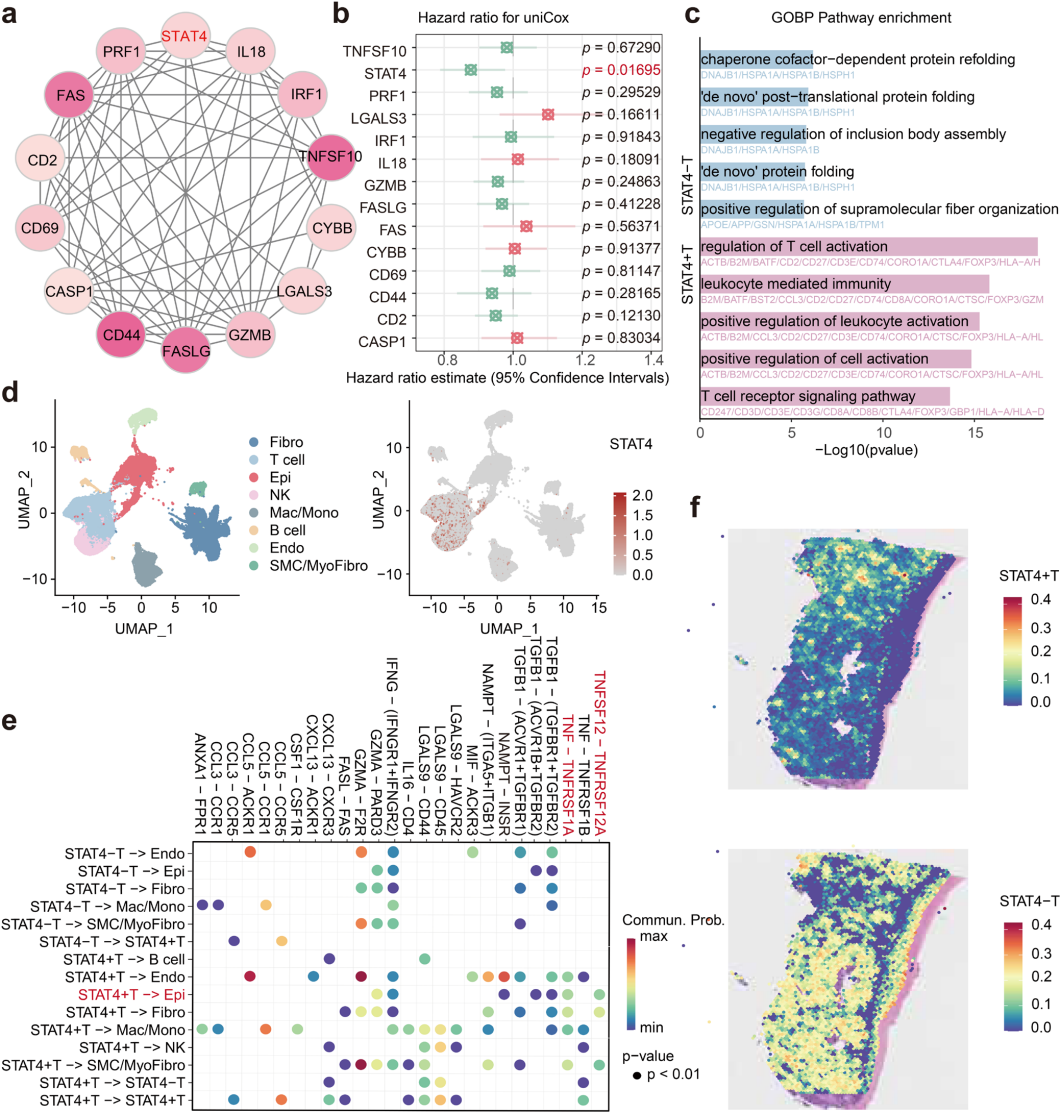
The analysis of *STAT4*. (a) Protein–protein interactions (PPI) of 14 PANoptosis‐related genes. (b) Univariate Cox regression showed that *STAT4* was a protective factor for OC. (c) The gene ontology (GO) BP analysis in *STAT4* ^+^ T cell and *STAT4* ^−^ T cell. (d) Single‐cell RNA‐seq of *STAT4*. (e) Ligand receptors in cell–cell communication. (f) Spatial transcriptomics RNA‐seq of *STAT4* ^+^ T cell and *STAT4* ^−^ T cell.

### 3.4 Analysis of Pathological Features Combined With PANoptosis‐Related Genes

By analysing the PANoptosis genes related to pathological features, a PPI network was performed on these genes, and 14 core genes were identified through Molecular Complex Detection (MCODE) screening, namely *STAT4*, *PRF1*, *FAS*, *CD2*, *CD69*, *CASP1*, *CD44*, *FASLG*, *GZMB*, *LGALS3*, *CYBB*, *TNFSF10*, *IRF1*, and *IL18* (Figure 6a). On this basis, Univariate Cox analysis was conducted, and Figure 6b showed that the *STAT4* gene was strongly protectively correlated with prognosis (*p* = 0.01695).

### 3.5 Cell Positioning and Functional Enrichment of *STAT4*

According to scRNA‐seq, *STAT4* was mainly expressed in T cells (Figure 6d). We further integrated more databases and obtained the same results. *STAT4* is mainly enriched in T cells, especially in tissue‐resident memory CD4+ T cells (Figure S7). Then, we explored the pathways related to *STAT4*. The GO BP results showed that positive regulation of leukocyte cell–cell adhesion, T cell receptor signalling pathway, positive regulation of leukocyte activation, leukocyte‐mediated immunity, and regulation of T cell activation were the main pathways enriched in *STAT4* ^+^ T cells (Figure 6c). Immune system process, regulation of biological process, biological regulation, and regulation of cellular process were the enrichment results obtained from GSEA analysis (Figure S8a). In addition, scMetabolism found that *STAT4* ^+^ T cells were enriched in pyruvate metabolism, pentose phosphate pathway, and others (Figure S8b). We also demonstrated the significant differences in the spatial distribution of *STAT4* ^−^ T cells and *STAT4* ^+^ T cells by stRNA‐seq (Figure 6f).

### 3.6 Analysis of Intercellular Interactions

Using spatial transcriptome data and correlation analysis, we found that the higher expression of *STAT4* is associated with higher PANoptotic scores in OC (Figure S9). The spatial distribution of *STAT4* and the CD3 Delta subunit of the T cell receptor complex (CD3D) shows high consistency. In addition, we calculated the relationship between the level of PANoptosis in OC cells and the nearest distance to *STAT4* ^+^ T cells. Overall, closer distances indicate higher levels of PANoptosis, which may suggest that *STAT4* ^+^ T cells may induce PANoptosis in nearby OC cells (Figure S10). To further investigate the impact of *STAT4* in OC, we conducted a cell–cell communication analysis using CellChat. In addition, essential ligand‐receptor pairs that may interact with *STAT4* ^−^ T cells and *STAT4* ^+^ T cells were identified (Figure 8c,d). From the details in Figure 6e, we found that *STAT4* ^+^ T cells targeted epithelial cells through multiple receptor‐ligand pairs, including the prominent TNFSF12‐TNFRSF12A and TNF‐TNFRSF1A interactions.

## 4 Discussion

The discovery of PANoptosis has opened up new avenues for treating numerous diseases, particularly cancer [43]. In our study, we harnessed a multi‐omics approach to delineate the correlation between PANoptosis and OC. We unravelled the critical role of PANoptosis in OC prognosis, offering a fresh perspective through pathological omics. The insights derived from our study may serve as a robust foundation for analysing clinical prognosis in OC, potentially paving the way for novel and more effective immunotherapeutic targets for OC patients.

The vital influence of PANoptosis, a novel form of PCD, in fine‐tuning anti‐tumour immunity in TME has been demonstrated in past research [44]. High levels of PANoptosis showed better predictive power of immunotherapy response in gastric cancer (GC) [45] and also showed high immune infiltration and high immunotherapy sensitivity in renal cell carcinoma [46]. The implications of these findings extend to the realm of OC, suggesting that further studies investigating the connection between PANoptosis and OC are paramount. Such a comprehension could facilitate a more comprehensive elucidation of intracellular death mechanisms associated with OC and inaugurate novel avenues of therapeutic intervention. Additionally, our analyses, including prognostic and immune infiltration investigations, suggested that a heightened PANoptosis score was associated with greater levels of immune infiltration and an improved prognosis in OC, consistent with prior studies on other diseases [47].

In light of the advanced staging of diagnosed OC patients, promptly and accurately discerning the prognosis has escalated into a cardinal challenge in the clinical sphere [48]. As the gold standard for clinical diagnosis of OC, pathological diagnosis plays a vital bridging role. Pathomics in predicting the prognosis of cancer has been applied to some extent in cancers such as hepatocellular carcinoma (HCC) [49] and colorectal cancer (CRC) [50]. In our investigation, we scrutinised pathological slices and leveraged CellProfiler and ResNet‐50 to extract PANoptosis‐related pathological features. Subsequently, 15 features strongly correlated with prognosis were identified to develop a prognostic model. Patients were categorised into high‐risk and low‐risk groups based on their individual risk scores. The low‐risk group of the train and validation set had a better prognosis, which suggested the model's robust predictive efficacy. There were also significant differences in immune infiltration, immunotherapy, and chemotherapy effects between the high‐ and low‐risk groups. Our results showed that critical immune cells such as T cells and macrophages have higher infiltration in the high‐risk group. The high‐risk group had a poor response to immunotherapy. Conversely, the low‐risk group displayed heightened sensitivity to a majority of drugs, except for Sepatronium bromide. Notably, Sepatronium bromide, an effective survivin inhibitor, impedes cell growth and proliferation and induces apoptosis within OC cells [51]. The combination of Sepatronium bromide and other drugs may have a better therapeutic effect on OC [52]. Choosing Sepatronium bromide or combination therapy may achieve better clinical efficacy for high‐risk populations.

Upon undertaking an intricate dissection of the identified 15 pathological prognostic features, we accentuated our exploration of the genes manifesting an appreciable association with PANoptosis, wherein *STAT4* emerged markedly as a key prognostic factor. *STAT4* is a member of the Signal Transduction and Transcription Activating Factor (STAT) gene family and can participate in various pathways that affect the occurrence and progression of cancer [53]. *STAT4*'s correlation with cell apoptosis, pyroptosis, and necroptotic apoptosis is well‐accepted, with several studies highlighting its role [54]. Also, studies have shown that *STAT4* can act as a necroptosis‐related gene and affect the prognosis of OC [55]. As research deepened, the correlation and specific mechanism between *STAT4* and PANoptosis were gradually clarified. Activating the IL12‐STAT4 signalling pathway can enhance Th1 cell function, thereby enhancing the anti‐tumour response of cytotoxic T lymphocytes in vivo [56]. In vitro analysis showed that *STAT4* can regulate CD8 T cell infiltration and increase the migration rate of CD8 T cells in OC tissue by inducing CCL5 secretion [57]. Our results also found that the *STAT4* gene was mainly expressed in T cells, and functional enrichment showed that *STAT4* was primarily involved in the process of regulating both T cell activation and the T cell receptor signalling pathway, consistent with the aforementioned research findings. In addition, the correlation between high expression of *STAT4* and high PANoptotic scores in OC is also mentioned in our conclusion. And closer distances to *STAT4* ^+^ T cells may indicate higher levels of PANoptosis of OC cells. According to CellChat, we found that *STAT4* ^+^ T cells can target epithelial cells through TNFSF12‐TNFRSF12A and TNF‐TNFRSF1A. As explored in the preceding components of our study, we found that epithelial cells had a higher PANoptosis score, and they were the main components of the ovaries. Implied by these findings, the *STAT4* gene may induce *STAT4* ^+^T cell infiltration through TNFSF12‐TNFRSF12A and TNF‐TNFRSF1A, further activating PANoptosis in epithelial cells and inhibiting cancer progression. Previous studies have confirmed that the tumour necrosis factor (TNF) can trigger the activation of various inflammatory factors, induce PANoptosis in cancer and thus kill cancer cells [58], which may be achieved through the STAT signalling pathway [59]. TNFSF12, TNFRSF12A, and TNFRSF1A, as ligands in the TNF family, also played a role in PCD, such as apoptosis [60], which can support our conclusion. These findings underscore the potential of PANPM to identify patients likely to benefit from therapies targeting the IL12‐STAT4 signalling pathway or TNF‐related pathways [61, 62]. For instance, patients with high *STAT4* expression could be prioritised for IL‐12‐based immunotherapies or TNF agonists in clinical trials, offering a precision medicine approach to OC treatment.

To integrate PANPM into clinical practice, the model can be incorporated into digital pathology platforms, enabling pathologists to compute risk scores from histological images during routine diagnosis [63, 64]. A PANPM‐based scoring system could serve as a diagnostic tool, guiding oncologists in stratifying patients and selecting targeted therapies. For example, combining PANPM scores with existing biomarkers, such as BRCA1/2 status, could enhance precision in treatment planning for both early‐ and late‐stage OC [65, 66]. Prospective clinical studies are needed to validate PANPM's utility in guiding treatment decisions.

While our study provides compelling evidence for the role of PANoptosis and *STAT4* ^+^ T cells in OC prognosis, several potential confounding factors warrant consideration. Firstly, the retrospective nature of the data sourced from public databases may introduce selection bias, as these datasets often lack comprehensive clinical details such as comorbidities, treatment adherence, or lifestyle factors, which could influence patient outcomes. Then, technical variability in pathological image processing might affect feature extraction accuracy. While we standardised methods using CellProfiler and ResNet‐50, batch effects could persist. Future studies should include external validation cohorts with harmonised protocols to mitigate this. Lastly, genetic and epigenetic heterogeneity among OC subtypes may differentially influence PANoptosis pathways. Our analysis pooled subtypes due to sample size limitations, potentially obscuring subtype‐specific effects. Subgroup analyses in larger cohorts could clarify these interactions.

Besides, the other important limitation of our investigation must be acknowledged. Our exploration confined itself to bioinformatics analyses and preliminary validation, signalling the need for more exhaustive biological experimentation. Most notably, the role of the keystone gene, *STAT4*, requires affirmation through rigorous cell experiments and comprehensive in vitro and in vivo analyses. Consequently, fortifying our propositions necessitates the accrual of more extensive clinical and experimental data.

In the future, we will consider prospectively collecting independent clinical cohorts from multiple centers and regions, including data from different levels of hospitals, different scanning devices, and different populations, to rigorously validate the applicability of the model. Design targeted clinical trials to prospectively validate the effectiveness and practicality of the model in real clinical workflows, ultimately driving its clinical translation.

In conclusion, our study pioneers a unique intersection of pathomics and PANoptosis, facilitating the formulation of a potent prognostic model for OC. This innovative convergence enriches the interpretability of OC prognosis. Importantly, we've identified that *STAT4* ^+^ T cells potentially mitigate OC progression via activation of PANoptosis in epithelial cells, implicating *STAT4* as a prospective biomarker for OC diagnosis and treatment.

## Author Contributions

**Yangyang Zhang:** conceptualization (equal), data curation (equal), formal analysis (equal), visualization (equal). **Mengqi Fang:** writing – original draft (lead), writing – review and editing (equal). **Xuanyu Wang:** data curation (equal), formal analysis (equal), visualization (equal). **Zhiwei Ying:** software (equal), visualization (equal). **Shufan Jiang:** writing – original draft (supporting). **Yangyuxiao Lu:** writing – original draft (supporting). **Keren He:** writing – original draft (supporting). **Shaocong Mo:** conceptualization (equal). **Fangfang Tao:** funding acquisition (lead), writing – review and editing (equal). **Ping Lü:** writing – review and editing (equal).

## Funding

This study is supported by National key R&D Program of China (Grant No. 2025YFG0100800) and National Natural Science Foundation of China (Grant No. 82474460).

## Conflicts of Interest

The authors declare no conflicts of interest.

## Supporting information


**Figure S1:** The criteria of inclusion and exclusion for pathological sections of patients with OC.
**Figure S2:** Image preprocessing in CellProfiler. Based on the ‘Unmix Colors’ module to separate H&E‐stained images and convert them into haematoxylin‐stained and eosin‐stained greyscale images, the H&E‐stained images were also converted to greyscale images using the ‘ColorToGray’ module.
**Figure S3:** (left) Pipeline 1. First, grayscale H&E, haematoxylin‐stained and eosin‐stained images were assessed by using the ‘MeasureImageQuality’ module with three types of features, including blur features, saturation features and threshold features. The intensity features were assessed by ‘MeasureImageIntensity’. Subsequently, ‘MeasureColocalization’ module measured the colocalisation and correlation between intensities in haematoxylin images and eosin images on a pixel‐by‐pixel basis. Next, ‘MeasureGranularity’ module outputted spectra of size measurements of the textures in three types of images. Finally, ‘MeasureTexture’ module measured the degree and nature of textures within three types of images to quantify their roughness and smoothness. (right) Pipeline 2. Haematoxylin‐stained images were segmented via ‘IdentifyPrimaryObjects’ module and ‘IdentifySecondaryObjects’ module. Quantitative image features of object shape, size, texture and pixel intensity distribution were further extracted via multiple modules, including measure models of ‘Object Intensity’, ‘Texture’ and ‘Object Size Shape’.
**Figure S4:** PANoptosis analysis and immune infiltration analysis in OC. (a) PANoptosis levels in ovary tissues from TCGA and GTEx databases. (b) The correlation between immune cell abundance and PANoptosis score according to CIBERSORT. (c) Expression of known cell markers in OC. (d) The CIBERSORT algorithm evaluates the immune infiltration levels of high and low PANoptotic.
**Figure S5:** Immune infiltration analysis in the high‐ and low‐risk groups. (a) The correlation between 15 pathological features and immune infiltration using CIBERSORT. (b) The correlation between 15 pathological features and immune infiltration using Xcell. (c) The correlation between the risk score and the abundance of immune cells according to Xcell. (d) ESTIMATE algorithm for evaluating immune infiltration levels in different risk groups.
**Figure S6:** Immune infiltration analysis and efficacy prediction in the high‐ and low‐risk groups. (a) The relationship between immune infiltration levels and risk score. (b) and (c) The prediction of immunotherapy efficacy according to TIDE. (d) The relationship between the risk score of OC patients and immune check‐related genes. (e) SubMap analysis of the two groups, with a smaller *p* value implied a more similarity of paired expression profiles. (f) Sensitivity of different risk groups to common chemotherapy drugs.
**Figure S7:** (a, b) UMAP visualisation of total cells from 18 datasets. (c) Enrichment scores of *STAT4* in each cell type are shown by UMAP, with a darker purple colour having higher scores. (d) UMAP visualisation of total type of T cells. (e) Enrichment scores of *STAT4* in each type of T cells.
**Figure S8:** The analysis of *STAT4*. (a) The enrichment results of *STAT4* obtained from GSEA analysis. (b) The number and strength of cell–cell communication in OC. (c) The functional enrichment of *STAT4* according to scMetabolism. (c) The cell–cell communication analysis of *STAT4* ^−^ T cells and *STAT4* ^+^ T cells.
**Figure S9:** (a) A H&E image of OC. (b) Correlation Analysis Between Apoptosis and *STAT4* according figure a. (c) A H&E image of OC. (d) Spatial transcriptomic analysis of PANoptosis scores. (e) Spatial transcriptomic analysis of *STAT4*'s cell distribution. (f) Correlation Analysis Between Apoptosis and *STAT4* according figure c.
**Figure S10:** (a) PANoptotic score of OC cells. (b) Spatial positioning of *STAT4*. (c) Spatial positioning of the CD3 Delta subunit of the T cell receptor complex (CD3D). (d) The relationship between the level of PANoptosis in OC cells and the nearest distance to *STAT4* ^+^ T cells.
**Table S1:** The 30 PANoptosis‐related pathomics prognostic features identified by univariate Cox analysis.
**Table S2:** The 180 PANoptosis‐related genes strongly associated with pathological features according to Spearman correlation analysis.
**Table S3:** The 14 hub genes identified by the MCODE plugin using default parameters (degree cutoff ≥ 2, node score cutoff ≥ 2, K‐core ≥ 2 and max depth = 100).

## Data Availability

In this study, publicly available datasets were collected. The OC data and normal ovarian data were downloaded from The Cancer Genome Atlas (TCGA) database (https://www.cancer.gov/ccg/research/genome‐sequencing/tcga) and the Genotype Tissue Expression Project (GTEx) database (https://www.gtexportal.org/home/index.html). The information of the PLCO cohort analysed during the current study is not publicly available for patient privacy purposes or authorization restrictions. Data access can be obtained through a reasonable request to Y.Z. (21301050051@m.fudan.edu.cn). Access to the data will be restricted to non‐commercial research which removes patient‐sensitive information. The H&E images and clinical information of the TCIA cohort were downloaded from the Cancer Imaging Archive (https://www.cancerimagingarchive.net/collection/ovarian‐bevacizumab‐response/). The GSE184880 single‐cell RNA sequencing (scRNA‐seq) dataset was available in the Gene Expression Omnibus (GEO) database (https://www.ncbi.nlm.nih.gov/geo/). Spatial location data and pathological images were obtained on the 10X Genomics website (https://support.10xgenomics.com/spatial‐gene‐expression/datasets) and Genomic Data Commons portal (GDC portal, https://portal.gdc.cancer.gov/). Custom codes were available on the GitHub repository (https://github.com/pigudog/PANPM).

## References

[1] R. L. Siegel , A. N. Giaquinto , and A. Jemal , “Cancer Statistics, 2024,” CA: A Cancer Journal for Clinicians 74, no. 1 (2024): 12–49. 38230766 10.3322/caac.21820

[2] D. K. Armstrong , R. D. Alvarez , F. J. Backes , et al., “NCCN Guidelines Insights: Ovarian Cancer, Version 3.2022,” Journal of the National Comprehensive Cancer Network 20, no. 9 (2022): 972–980. 36075393 10.6004/jnccn.2022.0047

[3] M. Kostrzanowski , G. Ziółkowski , and F. Dąbrowski , “Extreme Case of Surgical Port Metastasis in Ovarian Cancer,” Oncology (Williston Park, N.Y.) 38, no. 3 (2024): 110–114. 38517412 10.46883/2024.25921015

[4] N. Colombo , C. Sessa , A. du Bois , et al., “ESMO‐ESGO Consensus Conference Recommendations on Ovarian Cancer: Pathology and Molecular Biology, Early and Advanced Stages, Borderline Tumours and Recurrent Disease,” Annals of Oncology 30, no. 5 (2019): 672–705. 31046081 10.1093/annonc/mdz062

[5] D. S. McMeekin , T. Tillmanns , T. Chaudry , et al., “Timing Isn't Everything: An Analysis of When to Start Salvage Chemotherapy in Ovarian Cancer,” Gynecologic Oncology 95, no. 1 (2004): 157–164. 15385126 10.1016/j.ygyno.2004.07.008

[6] N. Colombo , D. Lorusso , and P. Scollo , “Impact of Recurrence of Ovarian Cancer on Quality of Life and Outlook for the Future,” International Journal of Gynecological Cancer 27, no. 6 (2017): 1134–1140. 28640766 10.1097/IGC.0000000000001023 PMC5499966

[7] K. Hänggi and B. Ruffell , “Cell Death, Therapeutics, and the Immune Response in Cancer,” Trends Cancer 9, no. 5 (2023): 381–396. 36841748 10.1016/j.trecan.2023.02.001 PMC10121860

[8] L. Qi and Z. Tang , “Prognostic Model Revealing Pyroptosis‐Related Signatures in Oral Squamous Cell Carcinoma Based on Bioinformatics Analysis,” Scientific Reports 14, no. 1 (2024): 6149. 38480853 10.1038/s41598-024-56694-y PMC10937718

[9] J. M. Gullett , R. E. Tweedell , and T. D. Kanneganti , “It's All in the PAN: Crosstalk, Plasticity, Redundancies, Switches, and Interconnectedness Encompassed by PANoptosis Underlying the Totality of Cell Death‐Associated Biological Effects,” Cells 11, no. 9 (2022): 1495. 35563804 10.3390/cells11091495 PMC9105755

[10] R. K. S. Malireddi , S. Kesavardhana , and T. D. Kanneganti , “ZBP1 and TAK1: Master Regulators of NLRP3 Inflammasome/Pyroptosis, Apoptosis, and Necroptosis (PAN‐Optosis),” Frontiers in Cellular and Infection Microbiology 9 (2019): 406. 31850239 10.3389/fcimb.2019.00406 PMC6902032

[11] Y. Wang and T. D. Kanneganti , “From Pyroptosis, Apoptosis and Necroptosis to PANoptosis: A Mechanistic Compendium of Programmed Cell Death Pathways,” Computational and Structural Biotechnology Journal 19 (2021): 4641–4657. 34504660 10.1016/j.csbj.2021.07.038 PMC8405902

[12] S. Oh , J. Lee , J. Oh , et al., “Integrated NLRP3, AIM2, NLRC4, Pyrin Inflammasome Activation and Assembly Drive PANoptosis,” Cellular & Molecular Immunology 20, no. 12 (2023): 1513–1526. 38008850 10.1038/s41423-023-01107-9 PMC10687226

[13] Y. Lou , D. Chen , Q. Gu , Q. Zhu , and H. Sun , “PANoptosis‐Related Molecule CASP2 Affects the Immune Microenvironment and Immunotherapy Response of Hepatocellular Carcinoma,” Heliyon 10, no. 6 (2024): e27302. 38509889 10.1016/j.heliyon.2024.e27302 PMC10950493

[14] D. Bolignano , F. Mattace‐Raso , C. Torino , et al., “Prognostic Models in the Clinical Arena,” Aging Clinical and Experimental Research 24, no. 4 (2012): 300–304. 23238306 10.1007/BF03325262

[15] C. S. N. Lam , A. A. Bharwani , E. H. Y. Chan , et al., “A Machine Learning Model for Colorectal Liver Metastasis Post‐Hepatectomy Prognostications,” Hepatobiliary Surgery and Nutrition 12, no. 4 (2023): 495–506. 37601005 10.21037/hbsn-21-453 PMC10432293

[16] X. Zhao , X. Zhang , F. Li , and C. Lu , “Exploration of the Prognostic Prediction Value of the PANoptosis‐Based Risk Score and Its Correlation With Tumor Immunity in Lung Adenocarcinoma,” Journal of Gene Medicine 26, no. 3 (2024): e3682. 38508210 10.1002/jgm.3682

[17] J. Yang , C. Wang , Y. Zhang , S. Cheng , Y. Xu , and Y. Wang , “A Novel Pyroptosis‐Related Signature for Predicting Prognosis and Evaluating Tumor Immune Microenvironment in Ovarian Cancer,” Journal of Ovarian Research 16, no. 1 (2023): 196. 37730669 10.1186/s13048-023-01275-2 PMC10512632

[18] Z. Wang , G. Chen , F. Dai , S. Liu , W. Hu , and Y. Cheng , “Identification and Verification of Necroptosis‐Related Gene Signature With Prognosis and Tumor Immune Microenvironment in Ovarian Cancer,” Frontiers in Immunology 13 (2022): 894718. 35812403 10.3389/fimmu.2022.894718 PMC9265217

[19] Z. Wang , M. A. Jensen , and J. C. Zenklusen , “A Practical Guide to the Cancer Genome Atlas (TCGA),” Methods in Molecular Biology 1418 (2016): 111–141. 27008012 10.1007/978-1-4939-3578-9_6

[20] GTEx Consortium , “The GTEx Consortium Atlas of Genetic Regulatory Effects Across Human Tissues,” Science 369, no. 6509 (2020): 1318–1330. 32913098 10.1126/science.aaz1776 PMC7737656

[21] J. Xu , Y. Fang , K. Chen , et al., “Single‐Cell RNA Sequencing Reveals the Tissue Architecture in Human High‐Grade Serous Ovarian Cancer,” Clinical Cancer Research 28, no. 16 (2022): 3590–3602. 35675036 10.1158/1078-0432.CCR-22-0296 PMC9662915

[22] C. W. Wang , C. C. Chang , M. A. Khalil , et al., “Histopathological Whole Slide Image Dataset for Classification of Treatment Effectiveness to Ovarian Cancer,” Scientific Data 9, no. 1 (2022): 25. 35087101 10.1038/s41597-022-01127-6 PMC8795433

[23] F. Song , C. G. Wang , J. Z. Mao , et al., “PANoptosis‐Based Molecular Subtyping and HPAN‐Index Predicts Therapeutic Response and Survival in Hepatocellular Carcinoma,” Frontiers in Immunology 14 (2023): 1197152. 37398672 10.3389/fimmu.2023.1197152 PMC10311484

[24] A. Marcolini , N. Bussola , E. Arbitrio , M. Amgad , G. Jurman , and C. Furlanello , “Histolab: A Python Library for Reproducible Digital Pathology Preprocessing With Automated Testing,” SoftwareX 20 (2022): 101237.

[25] A. E. Carpenter , T. R. Jones , M. R. Lamprecht , et al., “CellProfiler: Image Analysis Software for Identifying and Quantifying Cell Phenotypes,” Genome Biology 7, no. 10 (2006): R100. 17076895 10.1186/gb-2006-7-10-r100 PMC1794559

[26] A. C. Ruifrok and D. A. Johnston , “Quantification of Histochemical Staining by Color Deconvolution,” Analytical and Quantitative Cytology and Histology 23, no. 4 (2001): 291–299. 11531144

[27] R. Bala and R. Eschbach , eds., Spatial Color‐To‐Grayscale Transform Preserving Chrominance Edge Information. Color and Imaging Conference (Society of Imaging Science and Technology, 2004).

[28] K. He , X. Zhang , S. Ren , and J. Sun , eds., “Deep Residual Learning for Image Recognition,” in Proceedings of the IEEE Conference on Computer Vision and Pattern Recognition (IEEE, 2016), 770–778.

[29] R. Satija , J. A. Farrell , D. Gennert , A. F. Schier , and A. Regev , “Spatial Reconstruction of Single‐Cell Gene Expression Data,” Nature Biotechnology 33, no. 5 (2015): 495–502. 10.1038/nbt.3192 PMC4430369 25867923

[30] C. Hafemeister and R. Satija , “Normalization and Variance Stabilization of Single‐Cell RNA‐Seq Data Using Regularized Negative Binomial Regression,” Genome Biology 20, no. 1 (2019): 296. 31870423 10.1186/s13059-019-1874-1 PMC6927181

[31] I. Korsunsky , N. Millard , J. Fan , et al., “Fast, Sensitive and Accurate Integration of Single‐Cell Data With Harmony,” Nature Methods 16, no. 12 (2019): 1289–1296. 31740819 10.1038/s41592-019-0619-0 PMC6884693

[32] P. Zhang , B. Wen , J. Gong , et al., “Clinical Prognostication and Immunotherapy Response Prediction in Esophageal Squamous Cell Carcinoma Using the DNA Damage Repair‐Associated Signature,” Environmental Toxicology 39, no. 5 (2024): 2803–2816. 38287713 10.1002/tox.24155

[33] G. Yu , L. G. Wang , Y. Han , and Q. Y. He , “clusterProfiler: An R Package for Comparing Biological Themes Among Gene Clusters,” OMICS 16, no. 5 (2012): 284–287. 22455463 10.1089/omi.2011.0118 PMC3339379

[34] S. Jin , C. F. Guerrero‐Juarez , L. Zhang , et al., “Inference and Analysis of Cell‐Cell Communication Using CellChat,” Nature Communications 12, no. 1 (2021): 1088. 10.1038/s41467-021-21246-9 PMC7889871 33597522

[35] Y. Wu , S. Yang , J. Ma , et al., “Spatiotemporal Immune Landscape of Colorectal Cancer Liver Metastasis at Single‐Cell Level,” Cancer Discovery 12, no. 1 (2022): 134–153. 34417225 10.1158/2159-8290.CD-21-0316

[36] D. M. Cable , E. Murray , L. S. Zou , et al., “Robust Decomposition of Cell Type Mixtures in Spatial Transcriptomics,” Nature Biotechnology 40, no. 4 (2022): 517–526. 10.1038/s41587-021-00830-w PMC8606190 33603203

[37] M. Elosua‐Bayes , P. Nieto , E. Mereu , I. Gut , and H. Heyn , “SPOTlight: Seeded NMF Regression to Deconvolute Spatial Transcriptomics Spots With Single‐Cell Transcriptomes,” Nucleic Acids Research 49, no. 9 (2021): e50. 33544846 10.1093/nar/gkab043 PMC8136778

[38] P. Jiang , S. Gu , D. Pan , et al., “Signatures of T Cell Dysfunction and Exclusion Predict Cancer Immunotherapy Response,” Nature Medicine 24, no. 10 (2018): 1550–1558. 10.1038/s41591-018-0136-1 PMC6487502 30127393

[39] Y. Hoshida , J. P. Brunet , P. Tamayo , T. R. Golub , and J. P. Mesirov , “Subclass Mapping: Identifying Common Subtypes in Independent Disease Data Sets,” PLoS One 2, no. 11 (2007): e1195. 18030330 10.1371/journal.pone.0001195 PMC2065909

[40] D. Maeser , R. F. Gruener , and R. S. Huang , “oncoPredict: An R Package for Predicting In Vivo or Cancer Patient Drug Response and Biomarkers From Cell Line Screening Data,” Briefings in Bioinformatics 22, no. 6 (2021): bbab260. 34260682 10.1093/bib/bbab260 PMC8574972

[41] D. Zeng , Z. Ye , R. Shen , et al., “IOBR: Multi‐Omics Immuno‐Oncology Biological Research to Decode Tumor Microenvironment and Signatures,” Frontiers in Immunology 12 (2021): 687975. 34276676 10.3389/fimmu.2021.687975 PMC8283787

[42] Z. Zhang , C. Wang , W. Shi , Z. Wang , and W. Fu , “Construction of Store‐Operated Calcium Entry‐Related Gene Signature for Predicting Prognosis and Indicates Immune Microenvironment Infiltration in Stomach Adenocarcinomas,” Scientific Reports 14, no. 1 (2024): 22342. 39333689 10.1038/s41598-024-73324-9 PMC11436956

[43] X. Yi , J. Li , X. Zheng , et al., “Construction of PANoptosis Signature: Novel Target Discovery for Prostate Cancer Immunotherapy,” Molecular Therapy‐Nucleic Acids 33 (2023): 376–390. 37547288 10.1016/j.omtn.2023.07.010 PMC10400972

[44] J. Liu , M. Hong , Y. Li , D. Chen , Y. Wu , and Y. Hu , “Programmed Cell Death Tunes Tumor Immunity,” Frontiers in Immunology 13 (2022): 847345. 35432318 10.3389/fimmu.2022.847345 PMC9005769

[45] H. Pan , J. Pan , P. Li , and J. Gao , “Characterization of PANoptosis Patterns Predicts Survival and Immunotherapy Response in Gastric Cancer,” Clinical Immunology 238 (2022): 109019. 35470064 10.1016/j.clim.2022.109019

[46] Y. Xu , J. Hua , H. Que , et al., “Identification of PANoptosis‐Related Signature Reveals Immune Infiltration Characteristics and Immunotherapy Responses for Renal Cell Carcinoma,” BMC Cancer 24, no. 1 (2024): 292. 38439022 10.1186/s12885-024-12067-2 PMC10913266

[47] B. Zhang , B. Huang , X. Zhang , et al., “PANoptosis‐Related Molecular Subtype and Prognostic Model Associated With the Immune Microenvironment and Individualized Therapy in Pancreatic Cancer,” Frontiers in Oncology 13 (2023): 1217654. 37519797 10.3389/fonc.2023.1217654 PMC10382139

[48] P. Gaona‐Luviano , L. A. Medina‐Gaona , and K. Magaña‐Pérez , “Epidemiology of Ovarian Cancer,” Chinese Clinical Oncology 9, no. 4 (2020): 47. 32648448 10.21037/cco-20-34

[49] M. Chen , B. Zhang , W. Topatana , et al., “Classification and Mutation Prediction Based on Histopathology H&E Images in Liver Cancer Using Deep Learning,” Npj Precision Oncology 4 (2020): 14. 32550270 10.1038/s41698-020-0120-3 PMC7280520

[50] C. Saillard , R. Dubois , O. Tchita , et al., “Validation of MSIntuit as an AI‐Based Pre‐Screening Tool for MSI Detection From Colorectal Cancer Histology Slides,” Nature Communications 14, no. 1 (2023): 6695. 10.1038/s41467-023-42453-6 PMC10628260 37932267

[51] E. G. de Vries and S. de Jong , “Exploiting the Apoptotic Route for Cancer Treatment: A Single Hit Will Rarely Result in a Home Run,” Journal of Clinical Oncology 26, no. 32 (2008): 5151–5153. 18824700 10.1200/JCO.2008.18.3160

[52] L. J. Hou , X. X. Huang , L. N. Xu , et al., “YM155 Enhances Docetaxel Efficacy in Ovarian Cancer,” American Journal of Translational Research 10, no. 3 (2018): 696–708. 29636860 PMC5883111

[53] M. Cheng , Y. Liu , Y. Guo , et al., “Pan‐Cancer Analysis Reveals Signal Transducer and Activator of Transcription (STAT) Gene Family as Biomarkers for Prognostic Prediction and Therapeutic Guidance,” Frontiers in Genetics 14 (2023): 1120500. 36968603 10.3389/fgene.2023.1120500 PMC10034013

[54] X. Y. Liu , L. Y. Zhang , X. Y. Wang , et al., “STAT4‐Mediated Klotho Up‐Regulation Contributes to the Brain Ischemic Tolerance by Cerebral Ischemic Preconditioning via Inhibiting Neuronal Pyroptosis,” Molecular Neurobiology 61 (2023): 2336–2356. 37875707 10.1007/s12035-023-03703-2

[55] S. Nie , N. Ni , N. Chen , et al., “Development of a Necroptosis‐Related Gene Signature and the Immune Landscape in Ovarian Cancer,” Journal of Ovarian Research 16, no. 1 (2023): 82. 37095524 10.1186/s13048-023-01155-9 PMC10127035

[56] R. Lu , S. Wang , S. Jiang , et al., “Chrysin Enhances Antitumour Immunity Response Through the IL‐12‐STAT4 Signal Pathway in the B16F10 Melanoma Mouse Model,” Scandinavian Journal of Immunology 96, no. 2 (2022): e13177. 35484925 10.1111/sji.13177

[57] W. Wang , S. Liu , F. Lu , et al., “STAT4, a Potential Predictor of Prognosis, Promotes CD8 T‐Cell Infiltration in Ovarian Serous Carcinoma by Inducing CCL5 Secretion,” Oncology Reports 50, no. 1 (2023): 140. 37264954 10.3892/or.2023.8577 PMC10251369

[58] R. K. S. Malireddi , R. Karki , B. Sundaram , et al., “Inflammatory Cell Death, PANoptosis, Mediated by Cytokines in Diverse Cancer Lineages Inhibits Tumor Growth,” Immunohorizons 5, no. 7 (2021): 568–580. 34290111 10.4049/immunohorizons.2100059 PMC8522052

[59] M. Marchisio , P. M. Grimley , A. Di Baldassarre , E. Santavenere , and S. Miscia , “Novel Shift of Jak/Stat Signalling Characterizes the Protective Effect of Aurintricarboxylic Acid (ATA) From Tumor Necrosis Factor‐Alpha Toxicity in Human B Lymphocytes,” International Journal of Immunopathology and Pharmacology 17, no. 1 (2004): 5–14. 15000861 10.1177/039463200401700102

[60] F. X. Pimentel‐Muiños and B. Seed , “Regulated Commitment of TNF Receptor Signaling: A Molecular Switch for Death or Activation,” Immunity 11, no. 6 (1999): 783–793. 10626900 10.1016/s1074-7613(00)80152-1

[61] M. Koneru , T. J. Purdon , D. Spriggs , S. Koneru , and R. J. Brentjens , “IL‐12 Secreting Tumor‐Targeted Chimeric Antigen Receptor T Cells Eradicate Ovarian Tumors In Vivo,” Oncoimmunology 4, no. 3 (2015): e994446. 25949921 10.4161/2162402X.2014.994446 PMC4404840

[62] L. Zhao , G. Ji , X. Le , et al., “An Integrated Analysis Identifies STAT4 as a Key Regulator of Ovarian Cancer Metastasis,” Oncogene 36, no. 24 (2017): 3384–3396. 28114283 10.1038/onc.2016.487

[63] J. Breen , K. Allen , K. Zucker , et al., “Artificial Intelligence in Ovarian Cancer Histopathology: A Systematic Review,” Npj Precision Oncology 7, no. 1 (2023): 83. 37653025 10.1038/s41698-023-00432-6 PMC10471607

[64] M. Desbois , A. R. Udyavar , L. Ryner , et al., “Integrated Digital Pathology and Transcriptome Analysis Identifies Molecular Mediators of T‐Cell Exclusion in Ovarian Cancer,” Nature Communications 11, no. 1 (2020): 5583. 10.1038/s41467-020-19408-2 PMC7642433 33149148

[65] H. Cardenas , G. Jiang , J. Thomes Pepin , et al., “Interferon‐γ Signaling Is Associated With BRCA1 Loss‐of‐Function Mutations in High Grade Serous Ovarian Cancer,” Npj Precision Oncology 3 (2019): 32. 31840082 10.1038/s41698-019-0103-4 PMC6897992

[66] E. A. Stronach , A. Alfraidi , N. Rama , et al., “HDAC4‐Regulated STAT1 Activation Mediates Platinum Resistance in Ovarian Cancer,” Cancer Research 71, no. 13 (2011): 4412–4422. 21571862 10.1158/0008-5472.CAN-10-4111 PMC3130134

