# Supplementary material for: Development of a PANoptosis‐Related Pathomics Prognostic Model in Ovarian Cancer: A Multi‐Omics Study
Source: J Cell Mol Med. 2025 Nov 24;29(22):e70958. doi: 10.1111/jcmm.70958 (PMC12643048; doi:10.1111/jcmm.70958)
Supplement: Supplementary file 1 — Figure S1: The criteria of inclusion and exclusion for pathological sections of patients with OC. Figure S2: Image preprocessing in CellProfiler. Based on the ‘Unmix Colors’ module to separate H&E‐stained images and convert them into haematoxylin‐stained and eosin‐stained greyscale images, the H&E‐stained images were also converted to greyscale images using the ‘ColorToGray’ module. Figure S3: (left) Pipeline 1. First, grayscale H&E, haematoxylin‐stained and eosin‐stained images were assessed by using the ‘MeasureImageQuality’ module with three types of features, including blur features, saturation features and threshold features. The intensity features were assessed by ‘MeasureImageIntensity’. Subsequently, ‘MeasureColocalization’ module measured the colocalisation and correlation between intensities in haematoxylin images and eosin images on a pixel‐by‐pixel basis. Next, ‘MeasureGranularity’ module outputted spectra of size measurements of the textures in three types of images. Finally, ‘MeasureTexture’ module measured the degree and nature of textures within three types of images to quantify their roughness and smoothness. (right) Pipeline 2. Haematoxylin‐stained images were segmented via ‘IdentifyPrimaryObjects’ module and ‘IdentifySecondaryObjects’ module. Quantitative image features of object shape, size, texture and pixel intensity distribution were further extracted via multiple modules, including measure models of ‘Object Intensity’, ‘Texture’ and ‘Object Size Shape’. Figure S4: PANoptosis analysis and immune infiltration analysis in OC. (a) PANoptosis levels in ovary tissues from TCGA and GTEx databases. (b) The correlation between immune cell abundance and PANoptosis score according to CIBERSORT. (c) Expression of known cell markers in OC. (d) The CIBERSORT algorithm evaluates the immune infiltration levels of high and low PANoptotic. Figure S5: Immune infiltration analysis in the high‐ and low‐risk groups. (a) The correlation between 15 pathological feat [file JCMM-29-e70958-s001.docx]

Supplementary Fig. S1 The criteria of inclusion and exclusion for pathological sections of patients with OC.

_
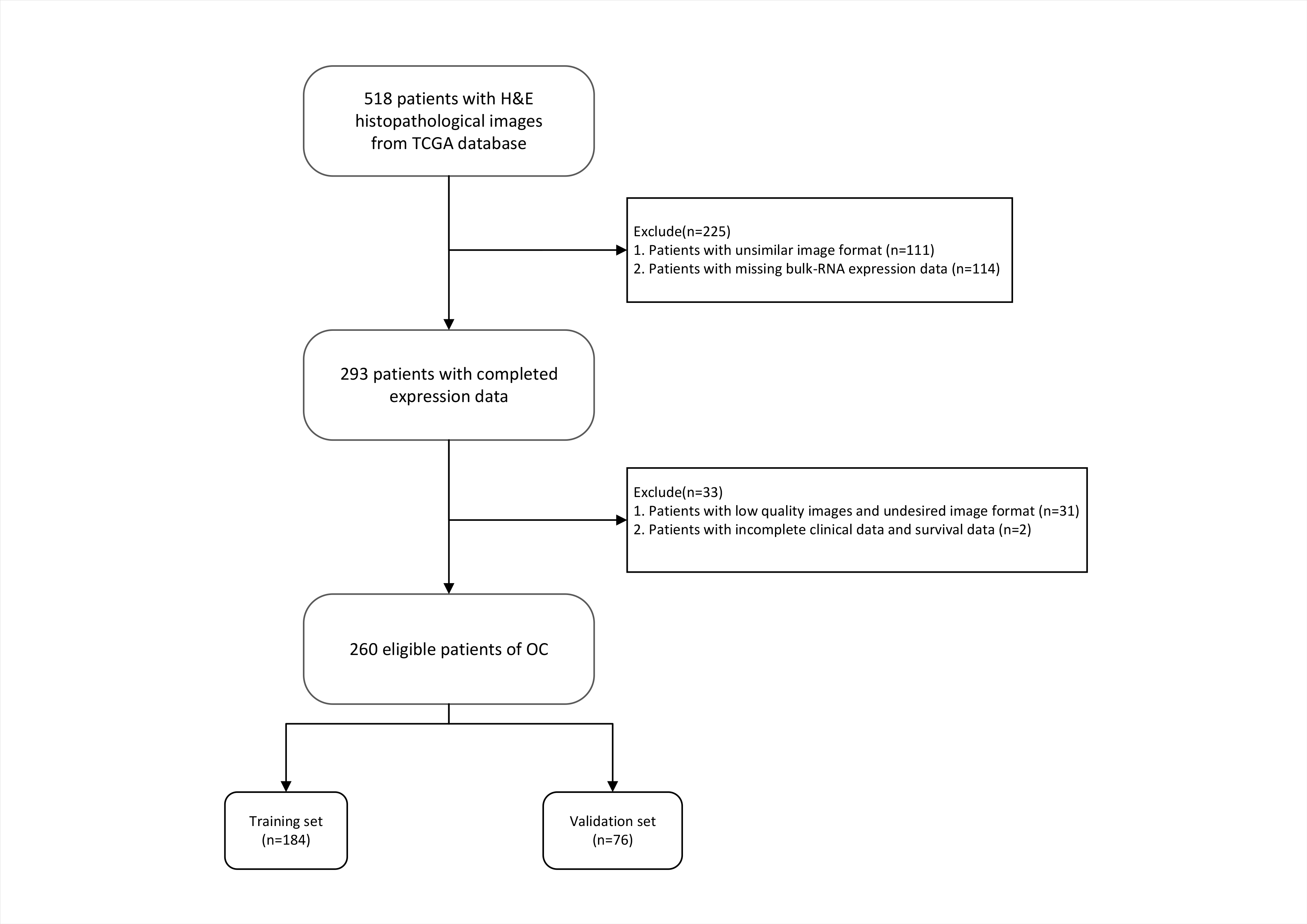
_

Supplementary Fig. S2 Image preprocessing in CellProfiler. Based on the “Unmix Colors” module to separate H&E-stained images and convert them into haematoxylin-stained and eosin-stained greyscale images, The H&E-stained images were also converted to greyscale images using the “ColorToGray” module.


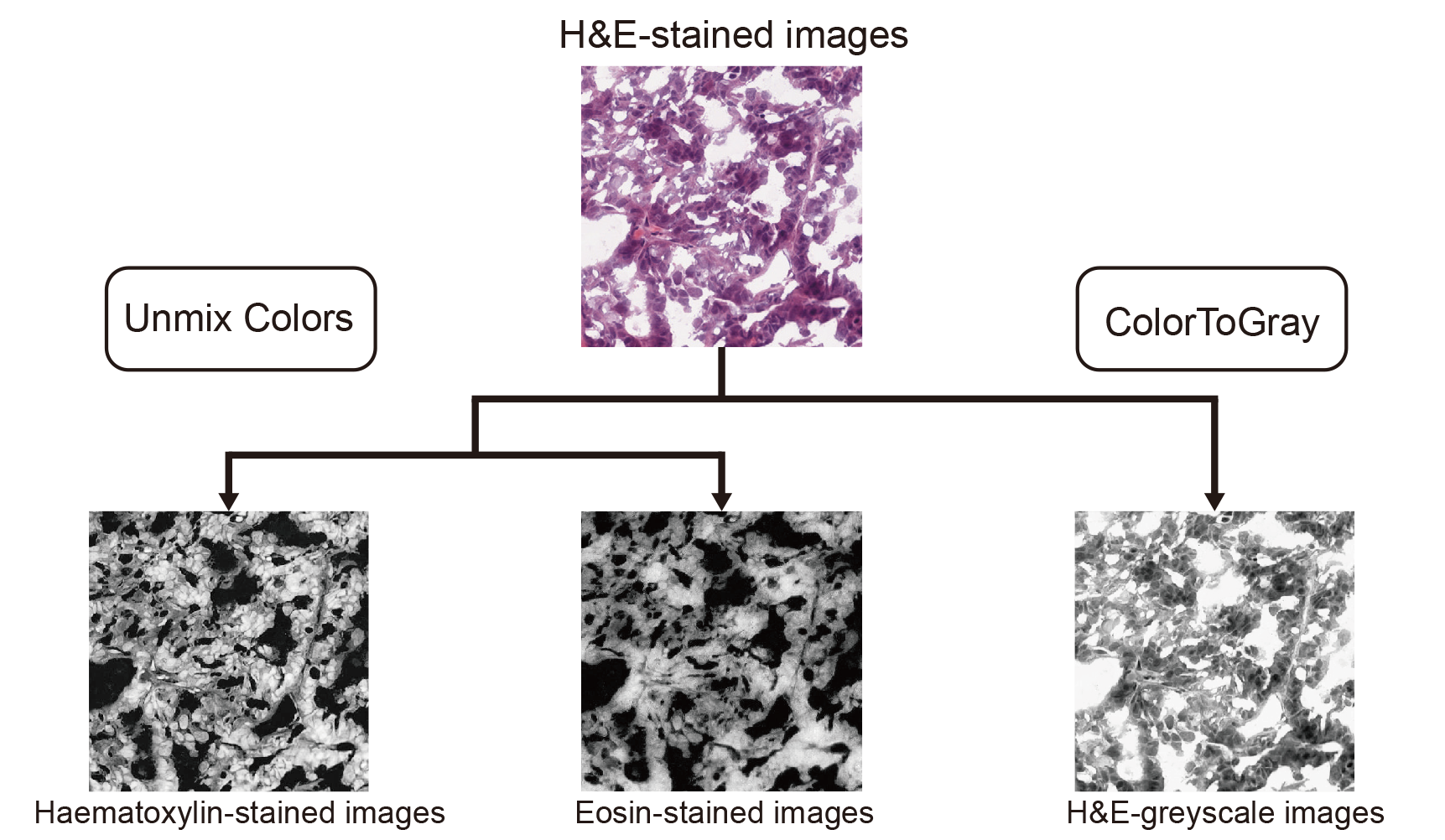


Supplementary Fig. S3 (left) Pipeline 1. First, grayscale H&E, haematoxylin-stained and eosin-stainded images were assessed by using the “MeasureImageQuality” module with three types of features, including blur features, saturation features and threshold features. The intensity features were assessed by “MeasureImageIntensity”. Subsequently, ‘MeasureColocalization’ module measured the colocalization and correlation between intensities in haematoxylin images and eosin images on a pixel-by-pixel basis. Next, ‘MeasureGranularity’ module outputed spectra of size measurements of the textures in three types of images. Finally, ‘MeasureTexture’ module measured the degree and nature of textures within three types of images to quantify their roughness and smoothness. (right) Pipeline 2. Haematoxylin-stained images were segmented via ‘IdentifyPrimaryObjects’module and‘IdentifySecondaryObjects’ module. Quantitative image features of object shape, size, texture, and pixel intensity distribution were further extracted via multiple modules, including measure models of ‘Object Intensity’, ‘Texture’, and ‘Object Size Shape’.


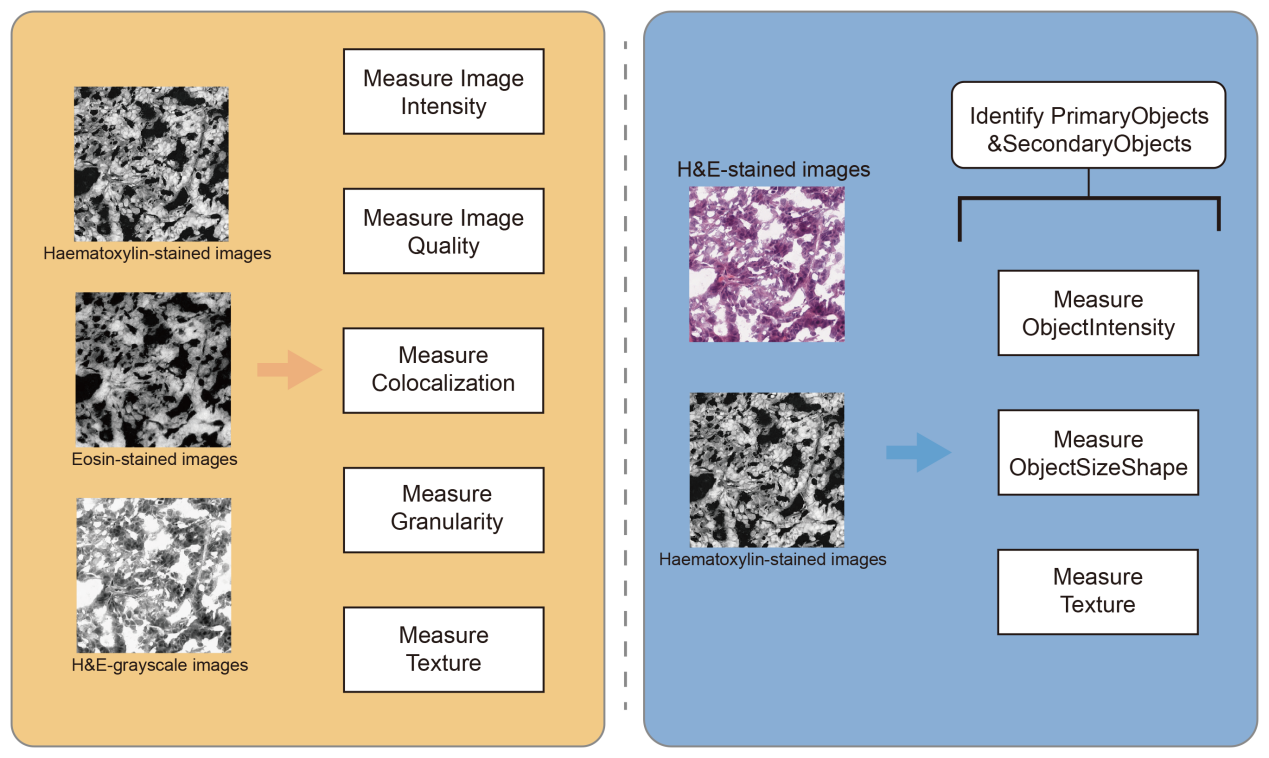


Supplementary Fig. S4 PANoptosis analysis and immune infiltration analysis in OC. (a) PANoptosis levels in ovary tissues from TCGA and GTEx databases. (b) The correlation between immune cell abundance and PANoptosis score according to CIBERSORT. (c) Expression of known cell markers in OC. (d) The CIBERSORT algorithm evaluates the immune infiltration levels of high and low PANoptotic. scores.
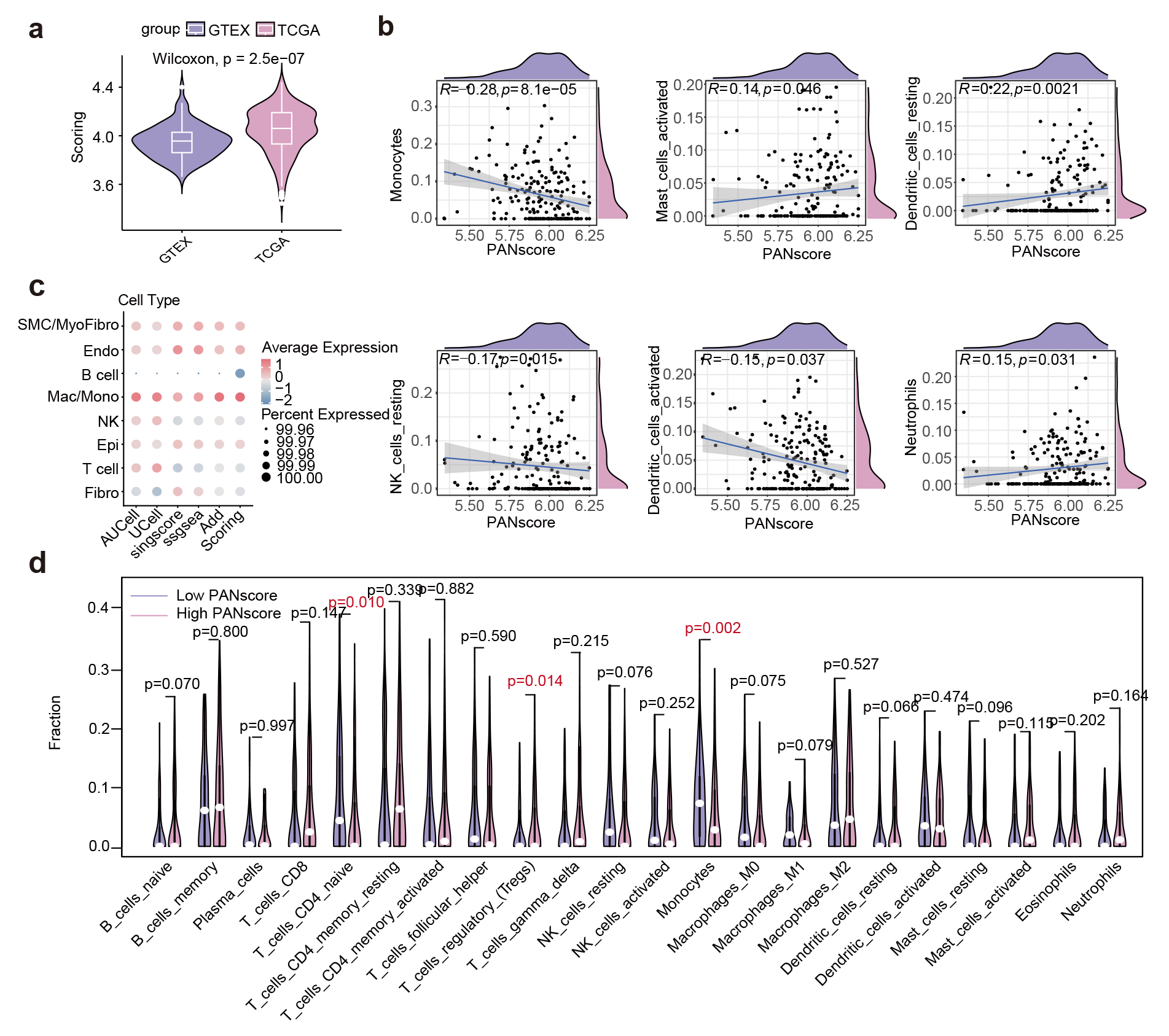


Supplementary Fig. S5 Immune infiltration analysis in the high- and low-risk groups. (a) The correlation between 15 pathological features and immune infiltration using CIBERSORT. (b) The correlation between 15 pathological features and immune infiltration using Xcell. (c) The correlation between the risk score and the abundance of immune cells according to Xcell. (d) ESTIMATE algorithm for evaluating immune infiltration levels in different risk groups.


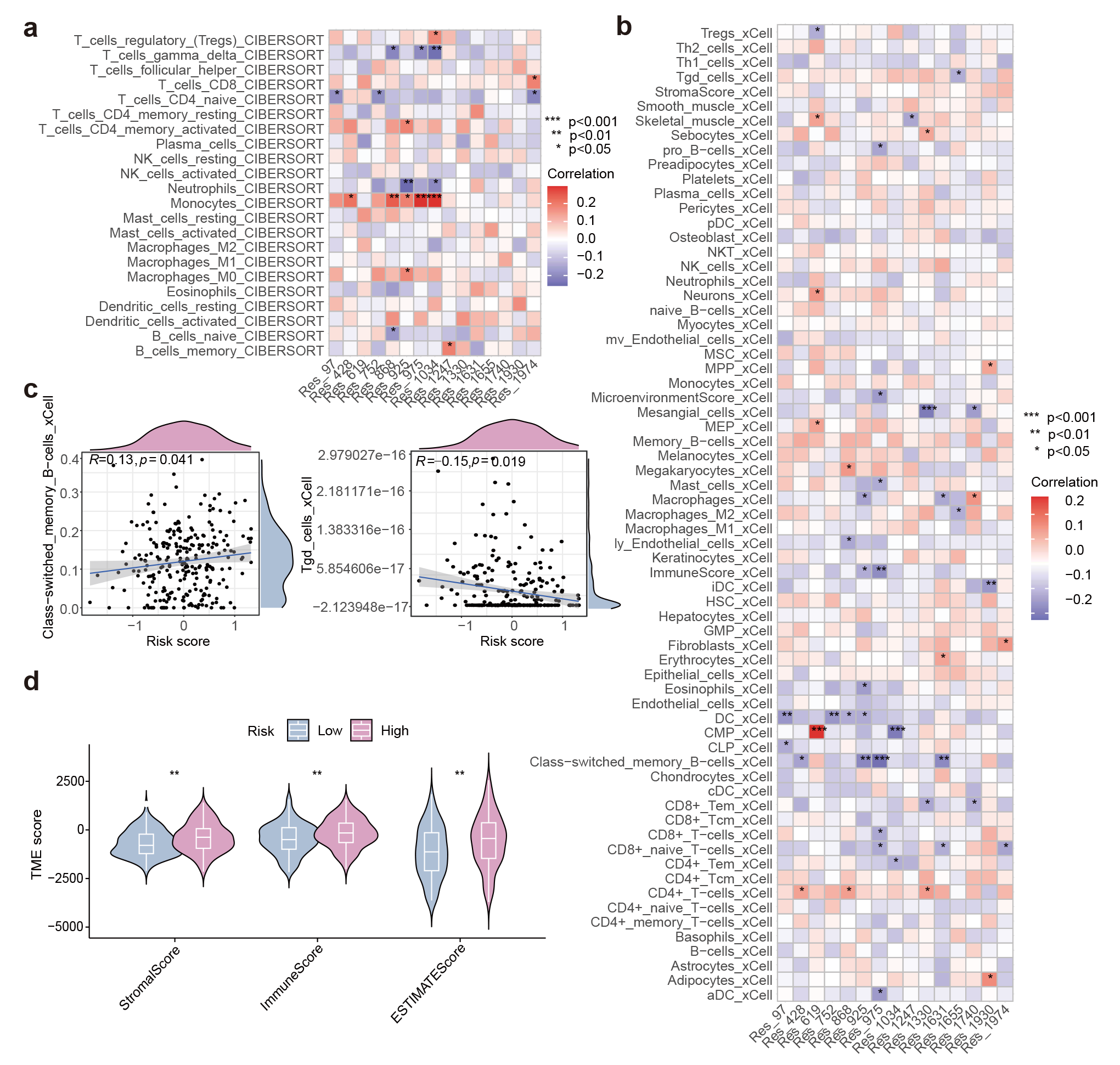


Supplementary Fig. S6 Supplementary Fig. S6 Immune infiltration analysis and efficacy prediction in the high- and low-risk groups. (a) The relationship between immune infiltration levels and risk score. (b) and (c) The prediction of immunotherapy efficacy according to TIDE. (d) The relationship between the risk score of OC patients and immune check-related genes. (e) SubMap analysis of the two groups, with a smaller p-value implied a more similarity of paired expression profiles. (f) Sensitivity of different risk groups to common chemotherapy drugs.


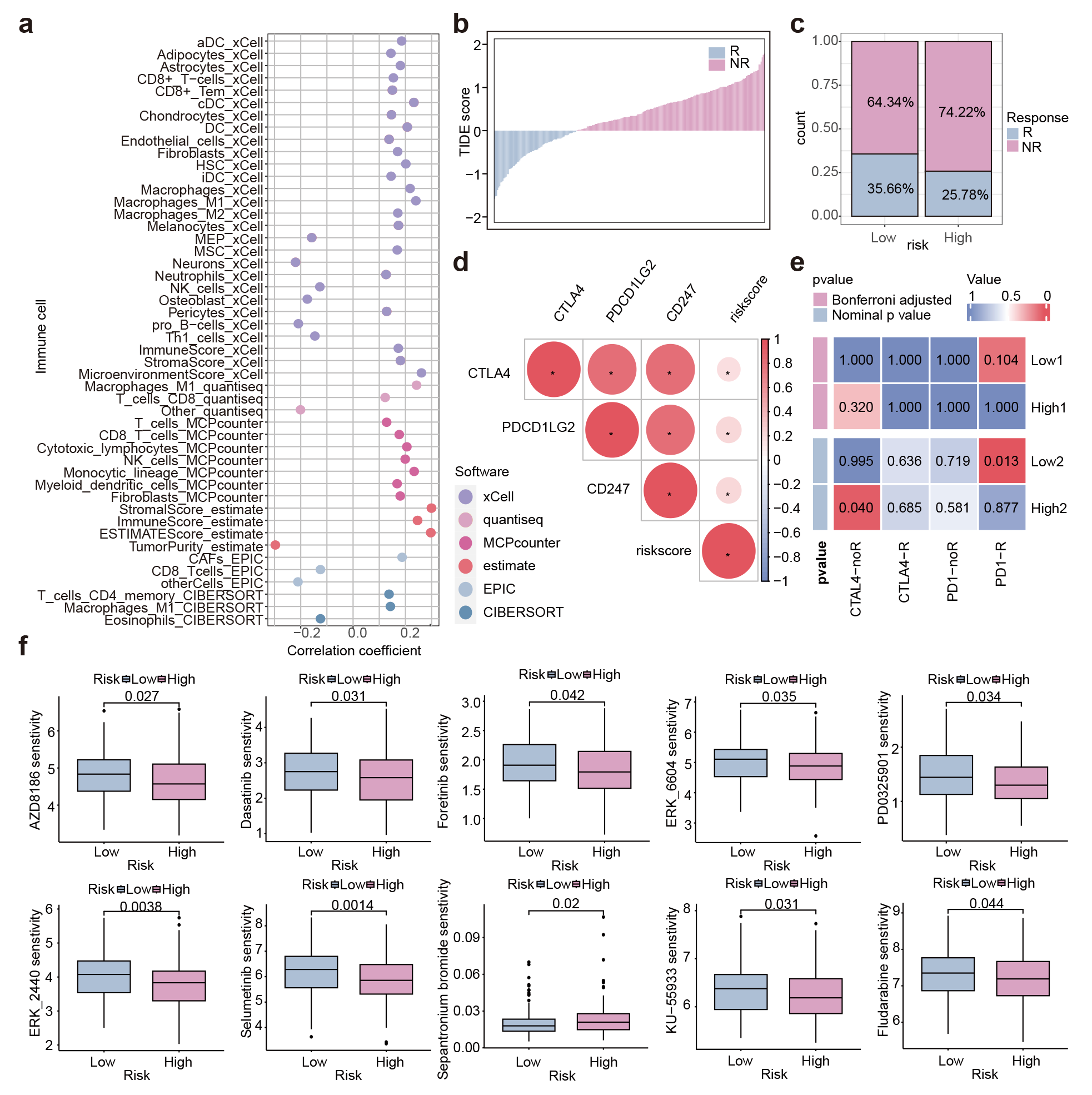


Supplementary Fig. S7 (a, b) UMAP visualization of total cells from 18 datasets. (c) Enrichment scores of STAT4 in each cell type are shown by UMAP, with a darker purple color having higher scores. (d) UMAP visualization of total type of T cells. (e) Enrichment scores of STAT4 in each type of T cells.


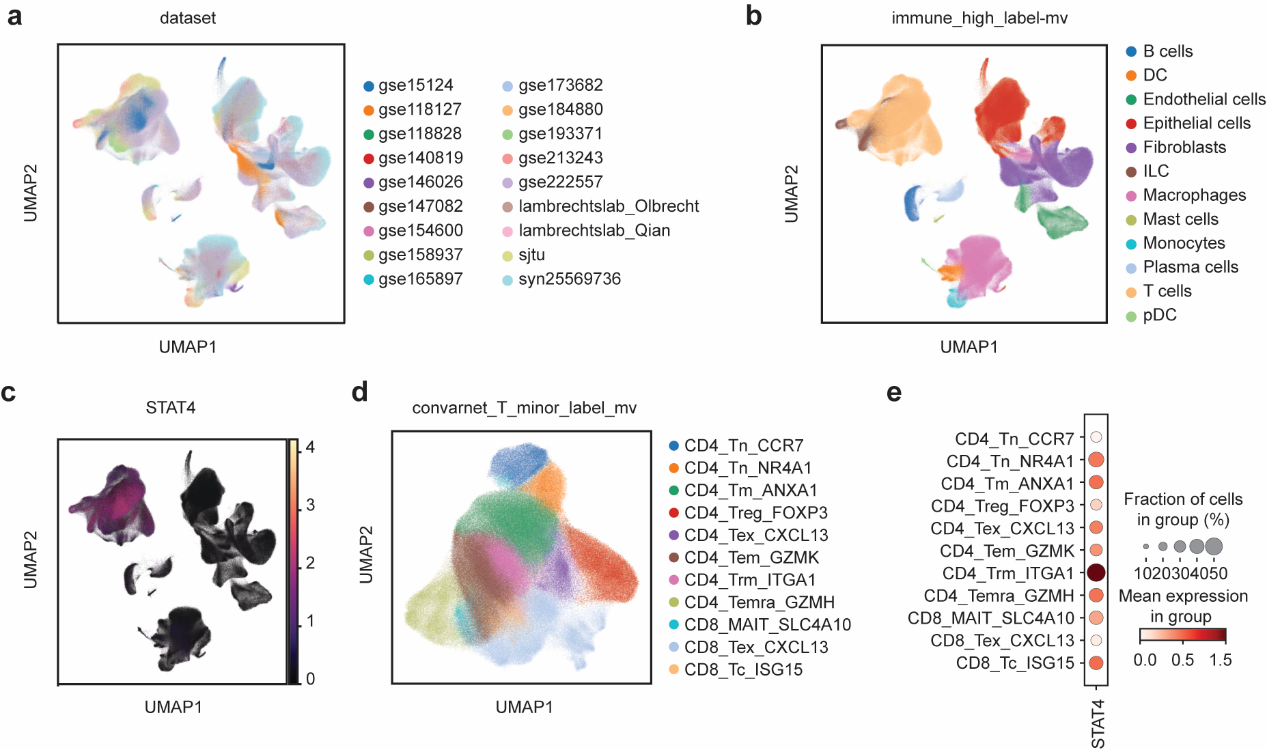


Supplementary Fig. S8 The analysis of *STAT4*. (a) The enrichment results of *STAT4* obtained from GSEA analysis. (b) The number and strength of cell-cell communication in OC. (c) The functional enrichment of *STAT4* according to scMetabolism. (c) The cell-cell communication analysis of *STAT4*^-^ T cells and *STAT4*^+^ T cells.

**
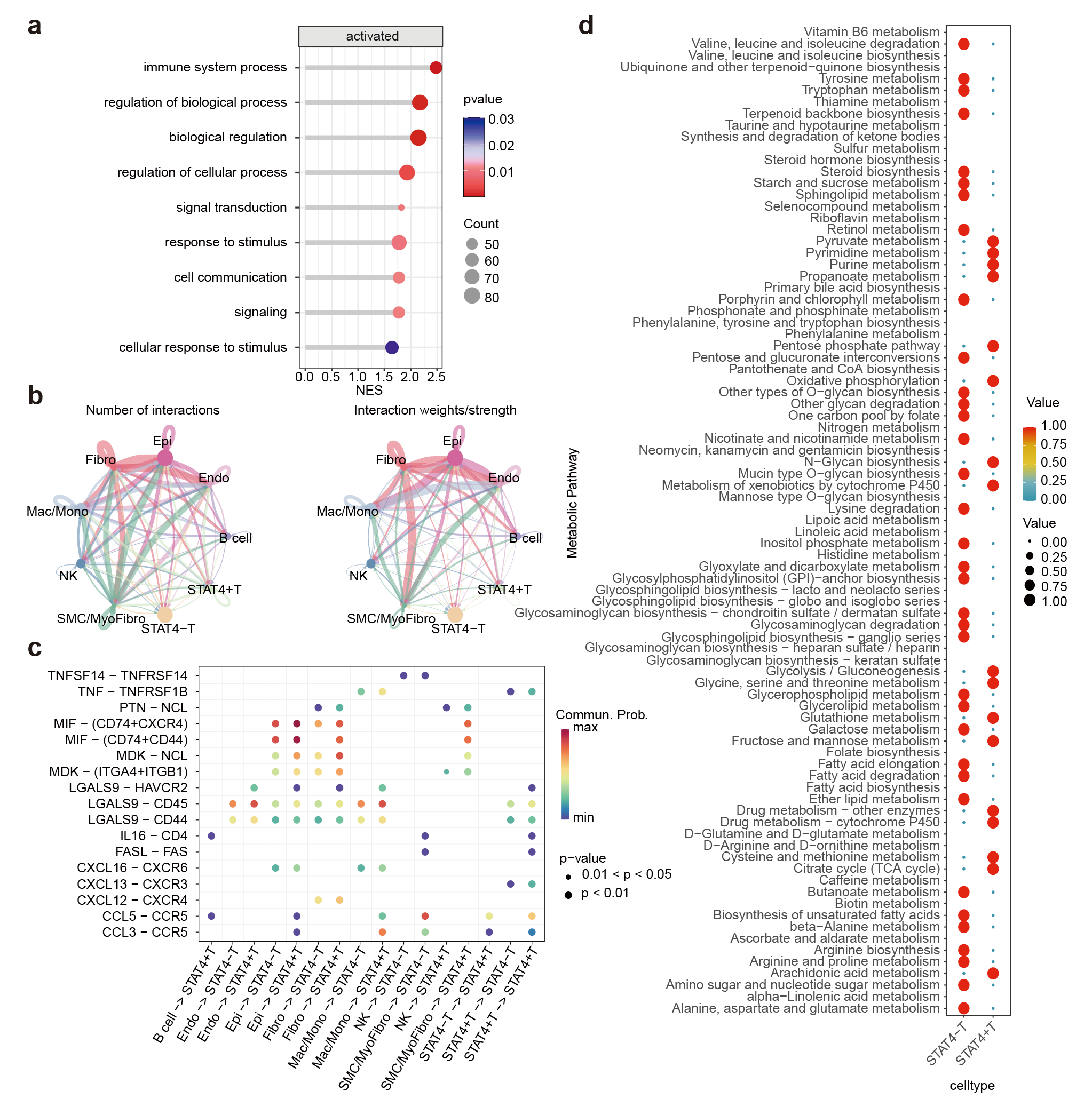
**

Supplementary Fig. S9 (a) A H&E image of OC. (b) Correlation Analysis Between Apoptosis and STAT4 according figure a. (c) A H&E image of OC. (d) Spatial transcriptomic analysis of PANoptosis scores. (e) Spatial transcriptomic analysis of STAT4’s cell distribution. (f) Correlation Analysis Between Apoptosis and STAT4 according figure c.

**
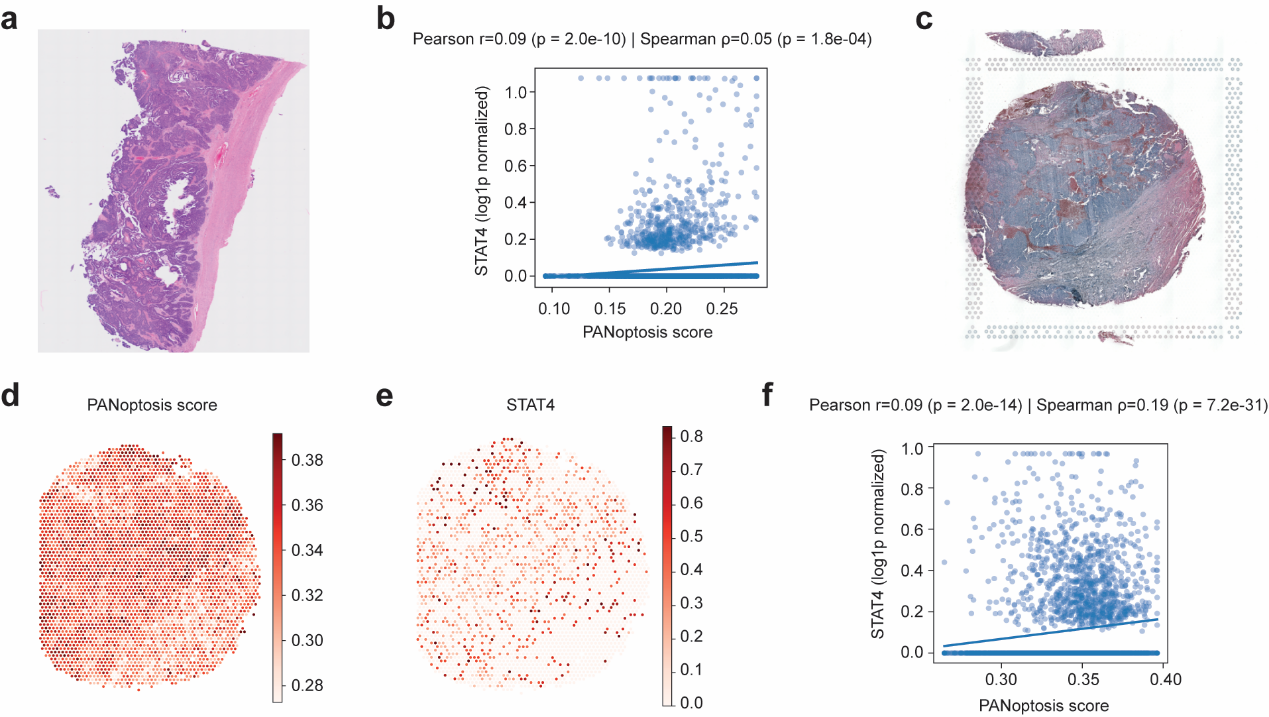
**

Supplementary Fig. S10 (a) PANoptotic score of OC cells. (b) Spatial positioning of STAT4. (c) Spatial positioning of the CD3 Delta subunit of the T cell receptor complex (CD3D). (d) The relationship between the level of PANoptosis in OC cells and the nearest distance to STAT4+ T cells.

**
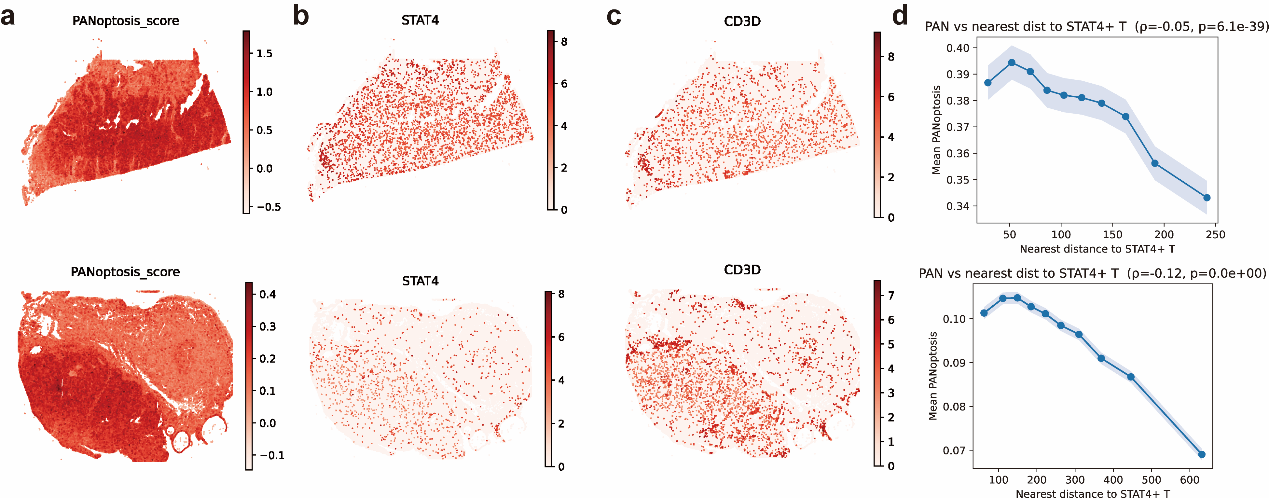
**

Supplementary Table S1 The 30 PANoptosis-related pathomics prognostic features identified by univariate Cox analysis.

| id | HR | HR.95L | HR.95H | pvalue |
| --- | --- | --- | --- | --- |
| Res_97 | 0.81 | 0.68 | 0.96 | 0.02 |
| Res_197 | 0.82 | 0.70 | 0.96 | 0.02 |
| Res_306 | 0.85 | 0.72 | 1.00 | 0.05 |
| Res_405 | 0.82 | 0.69 | 0.98 | 0.03 |
| Res_428 | 0.84 | 0.71 | 0.99 | 0.04 |
| Res_607 | 0.84 | 0.72 | 0.99 | 0.04 |
| Res_619 | 1.20 | 1.02 | 1.41 | 0.03 |
| Res_674 | 0.78 | 0.66 | 0.93 | 0.01 |
| Res_680 | 0.82 | 0.70 | 0.97 | 0.02 |
| Res_728 | 0.83 | 0.71 | 0.97 | 0.02 |
| Res_752 | 0.85 | 0.72 | 1.00 | 0.05 |
| Res_764 | 0.83 | 0.70 | 0.98 | 0.03 |
| Res_795 | 0.83 | 0.71 | 0.99 | 0.03 |
| Res_868 | 0.83 | 0.71 | 0.97 | 0.02 |
| Res_925 | 0.81 | 0.69 | 0.94 | 0.01 |
| Res_975 | 0.83 | 0.69 | 0.99 | 0.04 |
| Res_1034 | 0.80 | 0.68 | 0.94 | 0.01 |
| Res_1163 | 0.82 | 0.69 | 0.97 | 0.02 |
| Res_1247 | 0.82 | 0.69 | 0.99 | 0.04 |
| Res_1330 | 0.83 | 0.71 | 0.98 | 0.03 |
| Res_1631 | 0.81 | 0.68 | 0.96 | 0.02 |
| Res_1647 | 0.81 | 0.69 | 0.96 | 0.01 |
| Res_1655 | 1.23 | 1.06 | 1.43 | 0.01 |
| Res_1726 | 0.79 | 0.66 | 0.93 | 0.01 |
| Res_1740 | 0.82 | 0.69 | 0.97 | 0.02 |
| Res_1930 | 1.17 | 1.00 | 1.35 | 0.04 |
| Res_1974 | 0.80 | 0.66 | 0.97 | 0.02 |
| Res_2031 | 0.83 | 0.71 | 0.97 | 0.02 |
| Res_2038 | 0.85 | 0.73 | 0.99 | 0.04 |
| Res_2045 | 0.81 | 0.69 | 0.96 | 0.02 |

Supplementary Table S2 The 180 PANoptosis-related genes strongly associated with pathological features according to Spearman correlation analysis.

| feature | gene | correlation coefficient | text | pvalue |
| --- | --- | --- | --- | --- |
| Res_97 | BIRC3 | -0.21 | *** | 0.0008 |
| Res_97 | GNA15 | -0.21 | *** | 0.0006 |
| Res_97 | PSME2 | -0.21 | *** | 0.0009 |
| Res_97 | PIK3CG | -0.22 | *** | 0.0003 |
| Res_97 | FASLG | -0.24 | *** | 0.0001 |
| Res_97 | IRF1 | -0.21 | *** | 0.0006 |
| Res_97 | LGALS3 | -0.21 | *** | 0.0006 |
| Res_97 | CASP1 | -0.23 | *** | 0.0002 |
| Res_97 | PLCB2 | -0.24 | *** | 0.0001 |
| Res_97 | IL18 | -0.25 | *** | 0.0001 |
| Res_97 | CYBB | -0.21 | *** | 0.0005 |
| Res_97 | TAP1 | -0.21 | *** | 0.0006 |
| Res_97 | DPYD | -0.21 | *** | 0.0006 |
| Res_97 | CASP4 | -0.22 | *** | 0.0003 |
| Res_97 | PSMB10 | -0.26 | *** | < 0.0001 |
| Res_428 | CSF2RB | -0.26 | *** | < 0.0001 |
| Res_428 | PLA2G4C | -0.22 | *** | 0.0003 |
| Res_428 | PIK3CG | -0.22 | *** | 0.0004 |
| Res_428 | CD2 | -0.24 | *** | 0.0001 |
| Res_428 | FASLG | -0.22 | *** | 0.0003 |
| Res_428 | TLR4 | -0.22 | *** | 0.0003 |
| Res_428 | STAT4 | -0.22 | *** | 0.0003 |
| Res_428 | PIK3R5 | -0.22 | *** | 0.0004 |
| Res_428 | NLRP3 | -0.21 | *** | 0.0007 |
| Res_428 | CYBB | -0.24 | *** | 0.0001 |
| Res_428 | CD14 | -0.22 | *** | 0.0005 |
| Res_428 | PRF1 | -0.22 | *** | 0.0003 |
| Res_619 | TRAF5 | 0.23 | *** | 0.0002 |
| Res_619 | TRPM7 | 0.23 | *** | 0.0002 |
| Res_619 | USP21 | 0.21 | *** | 0.0007 |
| Res_619 | ATM | 0.21 | *** | 0.0007 |
| Res_619 | JMJD7-PLA2G4B | 0.22 | *** | 0.0004 |
| Res_619 | BCL2 | 0.23 | *** | 0.0002 |
| Res_619 | PLA2G4B | 0.23 | *** | 0.0002 |
| Res_752 | BIRC3 | -0.22 | *** | 0.0003 |
| Res_752 | PIK3CG | -0.22 | *** | 0.0005 |
| Res_752 | CD2 | -0.22 | *** | 0.0003 |
| Res_752 | FASLG | -0.27 | *** | < 0.0001 |
| Res_752 | TNFSF10 | -0.23 | *** | 0.0002 |
| Res_752 | IL1B | -0.21 | *** | 0.0009 |
| feature | gene | correlation coefficient | text | pvalue |
| Res_752 | IRAK2 | -0.22 | *** | 0.0004 |
| Res_752 | TLR4 | -0.21 | *** | 0.0007 |
| Res_752 | CASP1 | -0.22 | *** | 0.0004 |
| Res_752 | IL18 | -0.25 | *** | 0.0001 |
| Res_752 | NLRP3 | -0.22 | *** | 0.0003 |
| Res_752 | CYBB | -0.26 | *** | < 0.0001 |
| Res_752 | PRF1 | -0.21 | *** | 0.0006 |
| Res_868 | DCN | -0.31 | *** | < 0.0001 |
| Res_868 | HGF | -0.31 | *** | < 0.0001 |
| Res_868 | FAS | -0.23 | *** | 0.0003 |
| Res_868 | GNA15 | -0.25 | *** | < 0.0001 |
| Res_868 | MMP2 | -0.22 | *** | 0.0004 |
| Res_868 | HMOX1 | -0.26 | *** | < 0.0001 |
| Res_868 | CSF2RB | -0.31 | *** | < 0.0001 |
| Res_868 | PLA2G4C | -0.33 | *** | < 0.0001 |
| Res_868 | PIK3CG | -0.31 | *** | < 0.0001 |
| Res_868 | CD69 | -0.21 | *** | 0.0008 |
| Res_868 | PDGFRB | -0.25 | *** | 0.0001 |
| Res_868 | CD2 | -0.22 | *** | 0.0004 |
| Res_868 | FASLG | -0.25 | *** | 0.0001 |
| Res_868 | PLPPR4 | -0.27 | *** | < 0.0001 |
| Res_868 | IL1B | -0.21 | *** | 0.0006 |
| Res_868 | TLR4 | -0.30 | *** | < 0.0001 |
| Res_868 | CASP5 | -0.22 | *** | 0.0004 |
| Res_868 | PLCB2 | -0.22 | *** | 0.0003 |
| Res_868 | BCL2L10 | 0.25 | *** | 0.0001 |
| Res_868 | LUM | -0.32 | *** | < 0.0001 |
| Res_868 | PIK3R5 | -0.28 | *** | < 0.0001 |
| Res_868 | LY96 | -0.26 | *** | < 0.0001 |
| Res_868 | NLRP3 | -0.30 | *** | < 0.0001 |
| Res_868 | CYBB | -0.30 | *** | < 0.0001 |
| Res_868 | AVPR1A | -0.21 | *** | 0.0006 |
| Res_868 | CD14 | -0.28 | *** | < 0.0001 |
| Res_868 | PRF1 | -0.22 | *** | 0.0004 |
| Res_868 | BGN | -0.22 | *** | 0.0004 |
| Res_868 | DPYD | -0.23 | *** | 0.0002 |
| Res_925 | HGF | -0.25 | *** | 0.0001 |
| Res_925 | FAS | -0.27 | *** | < 0.0001 |
| Res_925 | CD44 | -0.25 | *** | 0.0001 |
| Res_925 | IFNGR1 | -0.25 | *** | < 0.0001 |
| Res_925 | PIK3CB | -0.23 | *** | 0.0002 |
| Res_925 | GNA15 | -0.22 | *** | 0.0003 |
| Res_925 | CYLD | -0.21 | *** | 0.0006 |
| feature | gene | correlation coefficient | text | pvalue |
| Res_925 | TRPM7 | -0.21 | *** | 0.0008 |
| Res_925 | HMOX1 | -0.25 | *** | 0.0001 |
| Res_925 | CSF2RB | -0.30 | *** | < 0.0001 |
| Res_925 | PLA2G4C | -0.26 | *** | < 0.0001 |
| Res_925 | PIK3CG | -0.31 | *** | < 0.0001 |
| Res_925 | CD69 | -0.27 | *** | < 0.0001 |
| Res_925 | IFNG | -0.22 | *** | 0.0003 |
| Res_925 | CD2 | -0.24 | *** | 0.0001 |
| Res_925 | FASLG | -0.28 | *** | < 0.0001 |
| Res_925 | ZBP1 | -0.22 | *** | 0.0003 |
| Res_925 | IL1B | -0.22 | *** | 0.0004 |
| Res_925 | BMP2 | -0.22 | *** | 0.0003 |
| Res_925 | IRAK2 | -0.22 | *** | 0.0005 |
| Res_925 | TLR4 | -0.33 | *** | < 0.0001 |
| Res_925 | CASP1 | -0.27 | *** | < 0.0001 |
| Res_925 | CASP5 | -0.25 | *** | < 0.0001 |
| Res_925 | PLCB2 | -0.21 | *** | 0.0006 |
| Res_925 | PIK3R5 | -0.29 | *** | < 0.0001 |
| Res_925 | JAK1 | -0.22 | *** | 0.0005 |
| Res_925 | NLRP3 | -0.31 | *** | < 0.0001 |
| Res_925 | CYBB | -0.30 | *** | < 0.0001 |
| Res_925 | FTH1 | -0.21 | *** | 0.0009 |
| Res_925 | TAP1 | -0.23 | *** | 0.0002 |
| Res_925 | CD14 | -0.26 | *** | < 0.0001 |
| Res_925 | PRF1 | -0.23 | *** | 0.0002 |
| Res_925 | DPYD | -0.30 | *** | < 0.0001 |
| Res_925 | GPX3 | -0.22 | *** | 0.0004 |
| Res_975 | DCN | -0.22 | *** | 0.0004 |
| Res_975 | HGF | -0.29 | *** | < 0.0001 |
| Res_975 | CD44 | -0.21 | *** | 0.0006 |
| Res_975 | GNA15 | -0.25 | *** | 0.0001 |
| Res_975 | HMOX1 | -0.27 | *** | < 0.0001 |
| Res_975 | CSF2RB | -0.36 | *** | < 0.0001 |
| Res_975 | GZMB | -0.25 | *** | < 0.0001 |
| Res_975 | PLA2G4C | -0.29 | *** | < 0.0001 |
| Res_975 | PIK3CG | -0.34 | *** | < 0.0001 |
| Res_975 | CD2 | -0.25 | *** | < 0.0001 |
| Res_975 | FASLG | -0.26 | *** | < 0.0001 |
| Res_975 | TLR4 | -0.34 | *** | < 0.0001 |
| Res_975 | CASP1 | -0.26 | *** | < 0.0001 |
| Res_975 | CASP5 | -0.25 | *** | < 0.0001 |
| Res_975 | PIK3R5 | -0.28 | *** | < 0.0001 |
| Res_975 | LY96 | -0.29 | *** | < 0.0001 |
| feature | gene | correlation coefficient | text | pvalue |
| Res_975 | NLRP3 | -0.30 | *** | < 0.0001 |
| Res_975 | CYBB | -0.32 | *** | < 0.0001 |
| Res_975 | TAP1 | -0.22 | *** | 0.0003 |
| Res_975 | CD14 | -0.30 | *** | < 0.0001 |
| Res_975 | TNFRSF10C | -0.21 | *** | 0.0007 |
| Res_975 | PRF1 | -0.26 | *** | < 0.0001 |
| Res_975 | DPYD | -0.26 | *** | < 0.0001 |
| Res_975 | PSMB8 | -0.20 | *** | 0.0010 |
| Res_975 | PSMB10 | -0.23 | *** | 0.0002 |
| Res_975 | PSMB9 | -0.22 | *** | 0.0005 |
| Res_1034 | HGF | -0.24 | *** | 0.0001 |
| Res_1034 | CD44 | -0.23 | *** | 0.0002 |
| Res_1034 | GNA15 | -0.22 | *** | 0.0005 |
| Res_1034 | CYLD | -0.22 | *** | 0.0004 |
| Res_1034 | HMOX1 | -0.24 | *** | 0.0001 |
| Res_1034 | CSF2RB | -0.29 | *** | < 0.0001 |
| Res_1034 | PIK3CG | -0.28 | *** | < 0.0001 |
| Res_1034 | FASLG | -0.22 | *** | 0.0004 |
| Res_1034 | RIPK3 | -0.23 | *** | 0.0002 |
| Res_1034 | PPT1 | -0.21 | *** | 0.0007 |
| Res_1034 | TLR4 | -0.28 | *** | < 0.0001 |
| Res_1034 | PLCB2 | -0.22 | *** | 0.0004 |
| Res_1034 | PIK3R5 | -0.27 | *** | < 0.0001 |
| Res_1034 | NLRP3 | -0.24 | *** | 0.0001 |
| Res_1034 | CYBB | -0.27 | *** | < 0.0001 |
| Res_1034 | CD14 | -0.22 | *** | 0.0004 |
| Res_1034 | DPYD | -0.20 | *** | 0.0010 |
| Res_1247 | GAS2 | 0.23 | *** | 0.0003 |
| Res_1330 | IL18 | -0.24 | *** | 0.0001 |
| Res_1631 | GZMB | -0.23 | *** | 0.0003 |
| Res_1631 | TICAM1 | -0.21 | *** | 0.0008 |
| Res_1631 | PRF1 | -0.22 | *** | 0.0003 |
| Res_1655 | CCND1 | 0.26 | *** | < 0.0001 |
| Res_1740 | FAS | -0.22 | *** | 0.0005 |
| Res_1740 | TGFBR3 | -0.22 | *** | 0.0003 |
| Res_1740 | FASLG | -0.22 | *** | 0.0004 |
| Res_1930 | VDAC3 | -0.24 | *** | 0.0001 |
| Res_1930 | HSP90AB1 | -0.22 | *** | 0.0005 |
| Res_1930 | TIMP3 | 0.22 | *** | 0.0005 |
| Res_1930 | PSMB5 | -0.25 | *** | 0.0001 |
| Res_1930 | LMNB1 | -0.27 | *** | < 0.0001 |
| Res_1930 | MACROH2A1 | -0.21 | *** | 0.0009 |
| Res_1930 | PSMF1 | -0.23 | *** | 0.0003 |
| feature | gene | correlation coefficient | text | pvalue |
| Res_1930 | RBCK1 | -0.22 | *** | 0.0004 |
| Res_1930 | PSMB4 | -0.22 | *** | 0.0004 |
| Res_1930 | FNTA | -0.21 | *** | 0.0006 |
| Res_1974 | HMOX1 | -0.24 | *** | 0.0001 |
| Res_1974 | CSF2RB | -0.22 | *** | 0.0003 |
| Res_1974 | GZMB | -0.21 | *** | 0.0008 |
| Res_1974 | PIK3CG | -0.24 | *** | 0.0001 |
| Res_1974 | CD2 | -0.23 | *** | 0.0002 |
| Res_1974 | FASLG | -0.23 | *** | 0.0003 |
| Res_1974 | PLCB2 | -0.22 | *** | 0.0004 |
| Res_1974 | PRF1 | -0.21 | *** | 0.0005 |

Supplementary Table S3 The 14 hub genes identified by the MCODE plugin using default parameters (degree cutoff ≥2, node score cutoff ≥2, K-core ≥2, and max depth =100).

| MCODE::Clusters | MCODE::Node Status | MCODE::Score | gene |
| --- | --- | --- | --- |
| Cluster 1 | Clustered | 10.64 | FAS |
| Cluster 1 | Clustered | 9.07 | IL18 |
| Cluster 1 | Clustered | 9.00 | LGALS3 |
| Cluster 1 | Clustered | 9.00 | CYBB |
| Cluster 1 | Clustered | 9.35 | GZMB |
| Cluster 1 | Clustered | 9.46 | IRF1 |
| Cluster 1 | Clustered | 10.86 | TNFSF10 |
| Cluster 1 | Clustered | 9.00 | STAT4 |
| Cluster 1 | Seed | 11.00 | CD44 |
| Cluster 1 | Clustered | 9.35 | PRF1 |
| Cluster 1 | Clustered | 8.84 | CD2 |
| Cluster 1 | Clustered | 9.23 | CD69 |
| Cluster 1 | Clustered | 10.64 | FASLG |
| Cluster 1 | Clustered | 8.80 | CASP1 |
